# Langevin Equations with Generalized Proportional Hadamard–Caputo Fractional Derivative

**DOI:** 10.1155/2021/6316477

**Published:** 2021-12-08

**Authors:** M. A. Barakat, Ahmed H. Soliman, Abd-Allah Hyder

**Affiliations:** ^1^Department of Computer Science, College of Al Wajh, University of Tabuk, Tabuk, Saudi Arabia; ^2^Department of Mathematics, Faculty of Sciences, Al-Azhar University, Assiut 71524, Egypt; ^3^King Khalid University, College of Science, Department of Mathematics, P.O. Box 9004, Abha 61413, Saudi Arabia; ^4^Department of Engineering Mathematics and Physics, Faculty of Engineering, Al-Azhar University, Cairo, Egypt

## Abstract

We look at fractional Langevin equations (FLEs) with generalized proportional Hadamard–Caputo derivative of different orders. Moreover, nonlocal integrals and nonperiodic boundary conditions are considered in this paper. For the proposed equations, the Hyres–Ulam (HU) stability, existence, and uniqueness (EU) of the solution are defined and investigated. In implementing our results, we rely on two important theories that are Krasnoselskii fixed point theorem and Banach contraction principle. Also, an application example is given to bolster the accuracy of the acquired results.

## 1. Introduction

In recent years, fractional calculus has gained great importance by numerous renowned mathematicians. The most essential feature of this topic is that it allows us to execute integrations and differentiations in any order, not necessarily integer ones Such a benefit has been encouraged by the applications in various areas, conceivably including fractal phenomena which appear in many sciences such as physics and engineering. Also, discovering the fractional derivatives and their generalizations was done by well-known mathematicians such as Riemann, Caputo, Hadamard, Euler, Liouville, Laplace, Laurent, and Fourier and was constantly the major path of research in the fractional calculus area. Moreover, these derivatives will provide us new chances to get generalized solutions of fractional differential equations [1–6]. It is worth to mention that the most important comprehensive treatments of differential equations with fractional order were initiated by Caputo [7] in 1969, and it was later called Caputo's derivative. There are many generalizations and modifications of this derivative [8–12], for example, the authors in [9] used Caputo's derivative to modify the Hadamard derivatives to a more beneficial concept that called Hadamard–Caputo derivatives (HCD). In 2019, Rahman et al. [12] introduced an integral form of Hadamard fractional derivatives which give generalized forms than the HCD and have shorted as GHCD. Many authors used HCD and their generalizations to study the existence, uniqueness, and stability of fractional differential equations [1, 6, 13, 14]. The researchers in [14] utilized the HCDs to present some results on the existence and stability for solutions of fractional Langevin equations with some conditions related to nonperiodic type boundary and nonlocal integral. Recently, Devi et al. [1] used two fixed point theorems due to Krasnoselskii and Banach as well as the HCDs of distinctive orders connected with nonlocal integral to establish the existence, uniqueness, and HU stability of solutions for fractional Langevin equations with nonperiodic boundary conditions.

According to varied literature, like [15–17], it has verified that the FLEs are perfectly mathematical models for single-file prevalence and the conduct of unshackled particles driven by internal noises. This motivates us to examine the solutions of the FLEs and their features. So, in the present work, we use the generalized proportional Hadamard–Caputo derivative to investigate the FLEs with nonlocal integrals and nonperiodic boundary conditions. For the proposed equations, the HU stability and EU of the solution are defined and analyzed. In implementing our results, we relied on two main fixed point theorems, namely, Krasnoselskii's theorem and Banach contraction operator. Furthermore, to reinforce the accuracy of the gained results, an application example is presented with adequate values for the parameters.

Our paper arranged as follows. In Section 2, we present all basic concepts related to the HCDs and their generalizations with some previous results which serve our results.  Section 3 devoted to present our main results which in turn was divided into two subsections, namely, 3.1, introduced to the existence and uniqueness of the solutions of the FLEs, and 3.2, prepared to study the stability results for the solutions of the FLEs.

## 2. Preliminaries

We use the set *𝔸ℂ*^*r*^[1, *e*] to refer to all absolutely continuous functions *ξ* such that its derivative of order (*r* − 1) is absolutely continuous on [1, *e*], where *r* ∈ *ℕ* and *e* is the renowned Euler's number.

**Definition 1 (see [18]).**

If *ξ* : [1, *∞*)⟶*ℝ* is both integrable and continuous, the one side fractional integral of Hadamard of *q*^th^-order is given by

(1) $$\begin{array}{c} {ℷ}^{q}\xi \left(\varpi \right)=\frac{1}{\Gamma \left(q\right)}\int_{1}^{\varpi }{\left(\ln\;\varpi -\ln\;u\right)}^{q-1}\frac{\xi \left(u\right)}{u}\text{d}u,\;q\in \mathbb{R},q>0. \end{array}$$

**Definition 2 (see [18]).**

The *q*^th^-order Hadamard fractional derivative is given by

(2) $$\begin{array}{c} D^{q}\xi \left(\varpi \right)=\frac{\left(\varpi \left(\text{d}/\text{d}\varpi \right)\right)}{\Gamma \left(m-q\right)}\int_{1}^{\varpi }{\left(\ln\;\varpi -\ln\;u\right)}^{m-q-1}\frac{\xi \left(u\right)}{u}\text{d}u, \end{array}$$

where *m*=[*q*]+1 and *q* > 0.

**Definition 3 (see [18]).**

The *q*^th^-order fractional HCD is given by

(3) Dℭℌqξϖ=1Γm−q∫1ϖln ϖ−ln um−q−1udduξuduu.

Rahman et al. [12] recently defined a generalized proportional Hadamard fractional integral of order *q*.

**Definition 4 (see [12]).**

The *q*^th^-order generalized proportional Hadamard fractional integral is given by

(4) $$\begin{array}{c} {ℷ}^{q,\varrho }\xi \left(\varpi \right)=\frac{1}{\varrho^{q}\Gamma \left(q\right)}\int_{1}^{\varpi }e^{\left(\left(\varrho -1\right)/\varrho \right)\ln\left(\varpi /u\right)}{\left(\ln\;\varpi -\ln\;u\right)}^{q-1}\frac{\xi \left(u\right)}{u}\text{d}u, \end{array}$$

where *q* > 1 and ϱ ∈ (0,1].

Finally, Jarad et al. [10] presented a broader range of fractional proportional integrals and derivatives.

**Definition 5 (see [10]).**

The *q*^th^-order of a broader range of the one side fractional proportional integrals is given by

(5) $$\begin{array}{c} {ℷ}^{q,\varrho ,\chi }\xi \left(\varpi \right)=\frac{1}{\varrho^{q}\Gamma \left(q\right)}\int_{1}^{\varpi }e^{\left(\left(\varrho -1\right)/\varrho \right)\left(\chi \left(\varpi \right)-\chi \left(u\right)\right)}{\left(\chi \left(\varpi \right)-\chi \left(u\right)\right)}^{q-1}\frac{\xi \left(u\right)}{u}\text{d}u, \end{array}$$

where *q* > 1, ϱ ∈ (0,1), and *χ* ∈ *C*[1, *e*].

**Definition 6 (see [10]).**

The *q*^th^-order of the one side of a broader range of fractional proportional derivative is given by

(6) $$\begin{array}{c} D^{q,\varrho ,\chi }\xi \left(\varpi \right)=\frac{D^{m,\varrho ,\chi }}{\Gamma \left(m-q\right)}\int_{1}^{\varpi }e^{\left(\left(\varrho -1\right)/\varrho \right)\left(\chi \left(\varpi \right)-\chi \left(u\right)\right)}{\left(\chi \left(\varpi \right)-\chi \left(u\right)\right)}^{m-q-1}\frac{\xi \left(u\right)}{u}\text{d}u, \end{array}$$

where *m*=[*q*]+1, ϱ ∈ (0,1), and *q* > 0.

**Definition 7 (see [10]).**

The *q*^th^-order of the one side of Caputo-broader range of fractional proportional derivative is given by

(7) Dℭq,ϱ,χξϖ=1Γm−q∫1ϖeϱ−1/ϱχϖ−χuχϖ−χum−q−1Dm,ϱ,χξuχ′udu,

where *m*=[*q*]+1, ϱ ∈ (0,1), and *q* > 0.

**Remark 1 .**

Obviously, if we put *χ*(*t*)=ln(*t*) in Definitions 5 and 7, we obtain the one side generalized proportional Caputo–Hadamard fractional integral and derivative, respectively.

By using Proposition 3.1, Proposition 4.1, and Remark 1.1 in [10], we obtain the following lemma.

**Lemma 1 (see [10]).**

Let *q*, *p* ∈ *ℂ* such that Re(*p*) > 0 and Re(*q*) > 0. Then, for any ϱ ∈ (0,1) and *m*=[Re(*q*)]+1, we have

- (ℷ^*q*,ϱ^*e*^((ϱ − 1)/ϱ)ln *t*^(ln *t*)^*p*−1^)(*ϖ*)=(Γ(*p*)/ϱ^*q*^Γ(*p*+*q*))*e*^((ϱ − 1)/ϱ)ln *ϖ*^(ln *ϖ*)^*q*+*p*−1^
- (*𝔇*^*q*,ϱ,*χ*^*e*^((ϱ − 1)/ϱ)ln *t*^(ln *t*)^*p*−1^)(*ϖ*)=(ϱ^*q*^Γ(*p*)/Γ(*p* − *q*))*e*^((ϱ − 1)/ϱ)ln *ϖ*^(ln *ϖ*)^*p*−*q*−1^

The following theorem is essential in implementing our results.

**Theorem 1 (see [8]).**

Let *K* be a nonempty convex, bounded, and closed subset of a Banach space *W*. Consider *J*_1_ and *J*_2_ are two operators from *K* to *W* such that

- *J*_1_*x*+*J*_2_*y* ∈ *K* for each *x*, *y* ∈ *K*
- *J*_1_ is continuous and compact
- *J*_2_ satisfies a contraction condition

Then, ∃*u* ∈ *K* such that *u*=*J*_1_*u*+*J*_2_*u*.

## 3. Main Results

This section comprises our new results for the fractional Langevin equations with generalized proportional Hadamard–Caputo derivative. By using Krasnoselskii and Banach fixed point theorems, we investigate the existence, uniqueness, and HU stability for these FLEs. Before we can show our results, we have to prove the following helpful lemmas.

**Lemma 2 .**

Let *q* ∈ *ℂ* such that Re(*q*) > 0, ϱ ∈ (0,1), *m*=[Re(*q*)]+1, *ξ* ∈ *L*_1_[1, *e*], and (ℷ^*q*,ϱ^*ξ*)(*ϖ*) ∈ *𝔸ℂ*^*m*^[1, *e*]. Then,

(8) ℷq,ϱDCHPq,ϱξϖ=ξϖ−∑r=1mDq−r,ϱξ1Γq−r+1ϱq−r·eϱ−1/ϱln ϖln ϖq−r.

Also, the fractional differential equation

(9) DCHPq,ϱξϖ=0

has a solution

(10) $$\begin{array}{c} \xi \left(\varpi \right)=e^{\left(\left(\varrho -1\right)/\varrho \right)\ln\;\varpi }\sum_{l=0}^{m-1}M_{l}{\left(\ln\;\varpi \right)}^{l}, \end{array}$$

where *M*_*l*_=(*𝔇*^*l*,ϱ^*ξ*(1)/Γ(*l*+1)ϱ^*l*^).

**Proof**

(11) $$\begin{array}{c} {ℷ}^{q,\varrho^{\mathrm{CHP}}}D^{q,\varrho }\xi \left(\varpi \right)=\frac{1}{\varrho^{q}\Gamma \left(q\right)}\int_{1}^{\varpi }e^{\left(\left(\varrho -1\right)/\varrho \right)\left(\ln\left(\varpi \right)-\ln\left(u\right)\right)}{\left(\ln\left(\varpi \right)-\ln\left(u\right)\right)}^{q-1}D^{m,\varrho }\left({ℷ}^{m-q,\varrho }\xi \left(u\right)\right)\frac{\text{d}u}{u}\\ =\frac{D^{1,\varrho }}{\varrho^{q+1}\Gamma \left(q\right)}\int_{1}^{\varpi }e^{\left(\left(\varrho -1\right)/\varrho \right)\left(\ln\left(\varpi \right)-\ln\left(u\right)\right)}{\left(\ln\left(\varpi \right)-\ln\left(u\right)\right)}^{q}D^{m,\varrho }\left({ℷ}^{m-q,\varrho }\xi \left(u\right)\right)\frac{\text{d}u}{u}\\ =D^{1,\varrho }\left[\frac{\varrho^{m}}{\varrho^{q+1}\Gamma \left(q-m+1\right)}\int_{1}^{\varpi }e^{\left(\left(\varrho -1\right)/\varrho \right)\left(\ln\left(\varpi \right)-\ln\left(u\right)\right)}{\left(\ln\left(\varpi \right)-\ln\left(u\right)\right)}^{q-m}\left({ℷ}^{m-q,\varrho }\xi \left(u\right)\right)\frac{\text{d}u}{u}\right.\\ \left.-\sum_{r=1}^{m}\frac{D^{q-r,\varrho }\xi \left(1\right)}{\Gamma \left(q-r+1\right)\varrho^{q-r}}\cdot e^{\left(\left(\varrho -1\right)/\varrho \right)\ln\;\varpi }{\left(\ln\;\varpi \right)}^{q-r}\right]\\ =D^{1,\varrho }\left[{ℷ}^{q-m+1,\varrho }\left({ℷ}^{m-q,\varrho }\xi \left(\varpi \right)\right)-\sum_{r=1}^{m}\frac{D^{q-r,\varrho }\xi \left(1\right)}{\Gamma \left(q-r+2\right)\varrho^{q-r+1}}\cdot e^{\left(\left(\varrho -1\right)/\varrho \right)\ln\;\varpi }{\left(\ln\;\varpi \right)}^{q-r+1}\right]\\ =D^{1,\varrho }\left[{ℷ}^{1,\varrho }\xi \left(\varpi \right)-\sum_{r=1}^{m}\frac{D^{q-r,\varrho }\xi \left(1\right)}{\Gamma \left(q-r+2\right)\varrho^{q-r+1}}\cdot e^{\left(\left(\varrho -1\right)/\varrho \right)\ln\;\varpi }{\left(\ln\;\varpi \right)}^{q-r+1}\right]\\ =\xi \left(u\right)-\sum_{r=1}^{m}\frac{D^{q-r,\varrho }\xi \left(1\right)}{\Gamma \left(q-r+1\right)\varrho^{q-r}}\cdot e^{\left(\left(\varrho -1\right)/\varrho \right)\ln\;\varpi }{\left(\ln\;\varpi \right)}^{q-r}. \end{array}$$

Also,

(12) $$\begin{array}{c} {ℷ}^{q,\varrho^{\mathrm{CHP}}}D^{q,\varrho }\xi \left(\varpi \right)={ℷ}^{q,\varrho }\left({ℷ}^{m-q,\varrho^{\mathrm{CHP}}}D^{m,\varrho }\xi \left(\varpi \right)\right)={ℷ}^{m,\varrho^{\mathrm{CHP}}}D^{m,\varrho }\xi \left(\varpi \right). \end{array}$$

By applying equation (11) in (12), we have

(13) $$\begin{array}{c} {ℷ}^{q,\varrho^{\mathrm{CHP}}}D^{q,\varrho }\xi \left(\varpi \right)=\xi \left(\varpi \right)-\sum_{l=0}^{m-1}\frac{D^{l,\varrho }\xi \left(1\right)}{\Gamma \left(l+1\right)\varrho^{l}}\cdot e^{\left(\left(\varrho -1\right)/\varrho \right)\ln\;\varpi }{\left(\ln\;\varpi \right)}^{l}, \end{array}$$

where the replacing of the integer *m* − *r* by *l* has been modified. Hence, the solution of the FDE (9) is given by

(14) $$\begin{array}{c} \xi \left(\varpi \right)=\sum_{l=0}^{m-1}\frac{D^{l,\varrho }\xi \left(1\right)}{\Gamma \left(l+1\right)\varrho^{l}}\cdot e^{\left(\left(\varrho -1\right)/\varrho \right)\ln\;\varpi }{\left(\ln\;\varpi \right)}^{l}. \end{array}$$

**Lemma 3 .**

The solution of the following fractional Langevin equation in the integral form

(15) $$\begin{array}{c} \left\{\begin{array}{c} {}^{\mathrm{CHP}}D^{q,\varrho }\left({}^{\mathrm{CHP}}D^{q,\varrho }+\lambda \right)z\left(t\right)=\xi \left(t,z\left(t\right)\right),\;t\in \left[1,e\right],\\ \begin{array}{c} z\left(1\right)=0,\\ z^{\prime }\left(1\right)=0,\\ z^{\prime \prime }\left(1\right)=0, \end{array}\\ {}^{\mathrm{CHP}}D^{q,\varrho }z\left(1\right)={ℷ}^{\vartheta ,\varrho }z\left(\eta \right),\;1<\eta <e,\\ {}^{\mathrm{CHP}}D^{q,\varrho }z\left(e\right)+lz\left(e\right)=0,\;l\in \mathbb{R}, \end{array}\right. \end{array}$$

is given by

(16) $$\begin{array}{c} z\left(t\right)=\frac{1}{\varrho^{p+q}\Gamma \left(p+q\right)}\int_{1}^{t}e^{\left(\left(\varrho -1\right)/\varrho \right)\left(\ln\;t-\ln\;u\right)}{\left(\ln\;t-\ln\;u\right)}^{p+q-1}\frac{\xi \left(u,z\left(u\right)\right)}{u}\text{d}u\\ -\frac{\lambda }{\varrho^{p}\Gamma \left(p\right)}\int_{1}^{t}e^{\left(\left(\varrho -1\right)/\varrho \right)\left(\ln\;t-\ln\;u\right)}{\left(\ln\;t-\ln\;u\right)}^{p-1}\frac{z\left(u\right)}{u}\text{d}u\\ +\frac{e^{\left(\left(\varrho -1\right)/\varrho \right)\ln\;t}{\left(\ln\;t\right)}^{p+1}}{\varrho^{p}\left(\lambda -l-\varrho^{p}\Gamma \left(p+2\right)\right)}\left(\frac{l-\lambda }{\varrho^{q}\Gamma \left(p+q\right)}\int_{1}^{e}e^{\left(\left(\varrho -1\right)/\varrho \right)\ln\;u}{\left(1-\ln\;u\right)}^{p+q-1}\frac{\xi \left(u,z\left(u\right)\right)}{u}\text{d}u\right.\\ +\frac{1}{\varrho^{q-p}\Gamma \left(q\right)}\int_{1}^{e}e^{\left(\left(\varrho -1\right)/\varrho \right)\ln\;u}{\left(1-\ln\;u\right)}^{q-1}\frac{\xi \left(u,z\left(u\right)\right)}{u}\text{d}u\\ -\frac{\lambda \left(\lambda -l\right)}{\Gamma \left(p\right)}\int_{1}^{e}e^{\left(\left(\varrho -1\right)/\varrho \right)\ln\;u}{\left(1-\ln\;u\right)}^{p-1}\frac{z\left(u\right)}{u}\text{d}u\\ +\left.\frac{l-\lambda +\varrho^{p}\Gamma \left(p+1\right)}{\varrho^{\vartheta }\Gamma \left(p+1\right)\Gamma \left(\vartheta \right)}\int_{1}^{\eta }e^{\left(\left(\varrho -1\right)/\varrho \right)\left(\ln\;\eta -\ln\;u\right)}{\left(\ln\;\eta -\ln\;u\right)}^{\vartheta -1}\frac{z\left(u\right)}{u}\text{d}u\right)\\ +\frac{e^{\left(\left(\varrho -1\right)/\varrho \right)\ln\;t}{\left(\ln\;t\right)}^{p}}{\varrho^{p+\vartheta }\Gamma \left(p+1\right)\Gamma \left(\vartheta \right)}\int_{1}^{\eta }e^{\left(\left(\varrho -1\right)/\varrho \right)\left(\ln\;\eta -\ln\;u\right)}{\left(\ln\;\eta -\ln\;u\right)}^{\vartheta -1}\frac{z\left(u\right)}{u}\text{d}u. \end{array}$$

**Proof**

Applying the generalized proportional Hadamard fractional integral ℷ^*q*,ϱ^ to equation (15), we get

(17) DCHPp,ϱzt+λzt=ℷq,ϱξt,zt+eϱ−1/ϱln tM0+M1ln t.

Repeating integration by using generalized proportional Hadamard fractional integral operator of order *p*, we obtain

(18) $$\begin{array}{c} z\left(t\right)={ℷ}^{q+p,\varrho }\xi \left(t,z\left(t\right)\right)-\lambda {ℷ}^{p,\varrho }z\left(t\right)+\left[M_{0}{ℷ}^{p,\varrho }\left(e^{\left(\left(\varrho -1\right)/\varrho \right)\ln\;t}\right)+M_{1}{ℷ}^{p,\varrho }\left(e^{\left(\left(\varrho -1\right)/\varrho \right)\ln\;t}\ln\;t\right)\right]. \end{array}$$

Since

(19) $$\begin{array}{c} {ℷ}^{p,\varrho }\left(e^{\left(\left(\varrho -1\right)/\varrho \right)\ln\;t}\right)=\frac{e^{\left(\left(\varrho -1\right)/\varrho \right)\ln\;t}{\left(\ln\;t\right)}^{p}}{\varrho^{p}\Gamma \left(p+1\right)}, \end{array}$$

(20) $$\begin{array}{c} {ℷ}^{p,\varrho }\left(e^{\left(\left(\varrho -1\right)/\varrho \right)\ln\;t}\ln\;t\right)=\frac{e^{\left(\left(\varrho -1\right)/\varrho \right)\ln\;t}{\left(\ln\;t\right)}^{p+1}}{\varrho^{p}\Gamma \left(p+2\right)}. \end{array}$$

Then, by substituting in (18) from (19) and (20), we get

(21) $$\begin{array}{c} z\left(t\right)={ℷ}^{q+p,\varrho }\xi \left(t,z\left(t\right)\right)-\lambda {ℷ}^{p,\varrho }z\left(t\right)+M_{0}\frac{e^{\left(\left(\varrho -1\right)/\varrho \right)\ln\;t}{\left(\ln\;t\right)}^{p}}{\varrho^{p}\Gamma \left(p+1\right)}+M_{1}\frac{e^{\left(\left(\varrho -1\right)/\varrho \right)\ln\;t}{\left(\ln\;t\right)}^{p+1}}{\varrho^{p}\Gamma \left(p+2\right)}+M_{2}e^{\left(\left(\varrho -1\right)/\varrho \right)\ln\;t}{\left(\ln\;t\right)}^{2}\\ +M_{3}e^{\left(\left(\varrho -1\right)/\varrho \right)\ln\;t}\left(\ln\;t\right)+M_{4}e^{\left(\left(\varrho -1\right)/\varrho \right)\ln\;t}, \end{array}$$

where *M*_0_, *M*_1_, *M*_2_, *M*_3_, and *M*_4_ are real constants.

By using the boundary condition, we get the following.

From *z*(1)=0⇒*M*_4_=0, *z*′(1)=0⇒*M*_3_=0, and *z*^″^(1)=0⇒*M*_2_=0.

Therefore,

(22) $$\begin{array}{c} z\left(t\right)={ℷ}^{q+p,\varrho }\xi \left(t,z\left(t\right)\right)-\lambda {ℷ}^{p,\varrho }z\left(t\right)+M_{0}\frac{e^{\left(\left(\varrho -1\right)/\varrho \right)\ln\;t}{\left(\ln\;t\right)}^{p}}{\varrho^{p}\Gamma \left(p+1\right)}+M_{1}\frac{e^{\left(\left(\varrho -1\right)/\varrho \right)\ln\;t}{\left(\ln\;t\right)}^{p+1}}{\varrho^{p}\Gamma \left(p+2\right)}. \end{array}$$

From the conditions,

(23) DCHPp,ϱz1=ℷϑ,ϱzη, 1<η<e,DCHPp,ϱze+lze=0, l∈ℝ.

By putting *t*=1 in (17), we get

(24) $$\begin{array}{c} M_{0}={ℷ}^{\vartheta ,\varrho }z\left(\eta \right). \end{array}$$

Also, by putting *t*=*e* in (17) and (22) and from equation (24), we have

(25) $$\begin{array}{c} M_{1}=\frac{\Gamma \left(p+2\right)}{\lambda -l-\varrho^{p}\Gamma \left(p+2\right)}\left(\frac{l-\lambda }{\varrho^{q}\Gamma \left(p+q\right)}\int_{1}^{e}e^{\left(\left(\varrho -1\right)/\varrho \right)\ln\;u}{\left(1-\ln\;u\right)}^{p+q-1}\frac{\xi \left(u,z\left(u\right)\right)}{u}\text{d}u\right.\\ +\frac{1}{\varrho^{q-p}\Gamma \left(q\right)}\int_{1}^{e}e^{\left(\left(\varrho -1\right)/\varrho \right)\ln\;u}{\left(1-\ln\;u\right)}^{p+q-1}\frac{\xi \left(u,z\left(u\right)\right)}{u}\text{d}u\\ -\frac{\lambda \left(\lambda -l\right)}{\Gamma \left(p\right)}\int_{1}^{e}e^{\left(\left(\varrho -1\right)/\varrho \right)\ln\;u}{\left(1-\ln\;u\right)}^{p-1}\frac{z\left(u\right)}{u}\text{d}u\\ +\left.\frac{l-\lambda +\varrho^{p}\Gamma \left(p+1\right)}{\varrho^{\vartheta }\Gamma \left(p+1\right)\Gamma \left(\vartheta \right)}\int_{1}^{\eta }e^{\left(\left(\varrho -1\right)/\varrho \right)\left(\ln\;\eta -\ln\;u\right)}{\left(\ln\;\eta -\ln\;u\right)}^{\vartheta -1}\frac{z\left(u\right)}{u}\text{d}u\right). \end{array}$$

Substituting in (22) from equations (24) and (25), we obtain

(26) $$\begin{array}{c} z\left(t\right)=\frac{1}{\varrho^{p+q}\Gamma \left(p+q\right)}\int_{1}^{t}e^{\left(\left(\varrho -1\right)/\varrho \right)\left(\ln\;t-\ln\;u\right)}{\left(\ln\;t-\ln\;u\right)}^{p+q-1}\frac{\xi \left(u,z\left(u\right)\right)}{u}\text{d}u\\ -\frac{\lambda }{\varrho^{p}\Gamma \left(p\right)}\int_{1}^{t}e^{\left(\left(\varrho -1\right)/\varrho \right)\left(\ln\;t-\ln\;u\right)}{\left(\ln\;t-\ln\;u\right)}^{p-1}\frac{z\left(u\right)}{u}\text{d}u\\ +\frac{e^{\left(\left(\varrho -1\right)/\varrho \right)\ln\;t}{\left(\ln\;t\right)}^{p+1}}{\varrho^{p}\left(\lambda -l-\varrho^{p}\Gamma \left(p+2\right)\right)}\left(\frac{l-\lambda }{\varrho^{q}\Gamma \left(p+q\right)}\int_{1}^{e}e^{\left(\left(\varrho -1\right)/\varrho \right)\ln\;u}{\left(1-\ln\;u\right)}^{p+q-1}\frac{\xi \left(u,z\left(u\right)\right)}{u}\text{d}u\right.\\ +\frac{1}{\varrho^{q-p}\Gamma \left(q\right)}\int_{1}^{e}e^{\left(\left(\varrho -1\right)/\varrho \right)\ln\;u}{\left(1-\ln\;u\right)}^{q-1}\frac{\xi \left(u,z\left(u\right)\right)}{u}\text{d}u\\ -\frac{\lambda \left(\lambda -l\right)}{\Gamma \left(p\right)}\int_{1}^{e}e^{\left(\left(\varrho -1\right)/\varrho \right)\ln\;u}{\left(1-\ln\;u\right)}^{p-1}\frac{z\left(u\right)}{u}\text{d}u\\ \left.+\frac{l-\lambda +\varrho^{p}\Gamma \left(p+1\right)}{\varrho^{\vartheta }\Gamma \left(p+1\right)\Gamma \left(\vartheta \right)}\int_{1}^{\eta }e^{\left(\left(\varrho -1\right)/\varrho \right)\left(\ln\;\eta -\ln\;u\right)}{\left(\ln\;\eta -\ln\;u\right)}^{\vartheta -1}\frac{z\left(u\right)}{u}\text{d}u\right)\\ +\frac{e^{\left(\left(\varrho -1\right)/\varrho \right)\ln\;t}{\left(\ln\;t\right)}^{p}}{\varrho^{p+\vartheta }\Gamma \left(p+1\right)\Gamma \left(\vartheta \right)}\int_{1}^{\eta }e^{\left(\left(\varrho -1\right)/\varrho \right)\left(\ln\;\eta -\ln\;u\right)}{\left(\ln\;\eta -\ln\;u\right)}^{\vartheta -1}\frac{z\left(u\right)}{u}\text{d}u. \end{array}$$

Suppose that the space (*𝒞*[1, *e*], ‖.‖) is a Bananch space and its norm is defined by ‖*𝔣*‖=sup_*t*∈[1, *e*]_*𝔣*(*𝔱*) such that, for each *𝔣* ∈ *𝒞*[1, *e*], it implies that *𝔣* : [1, *e*]⟶*ℝ* is continuous.

Let us start by defining an operator *𝔍* : *𝒞*[1, *e*]⟶*𝒞*[1, *e*] in order for

(27) $$\begin{array}{c} Jz\left(t\right)=\frac{1}{\varrho^{p+q}\Gamma \left(p+q\right)}\int_{1}^{t}e^{\left(\left(\varrho -1\right)/\varrho \right)\left(\ln\;t-\ln\;u\right)}{\left(\ln\;t-\ln\;u\right)}^{p+q-1}\frac{\xi \left(u,z\left(u\right)\right)}{u}\text{d}u\\ -\frac{\lambda }{\varrho^{p}\Gamma \left(p\right)}\int_{1}^{t}e^{\left(\left(\varrho -1\right)/\varrho \right)\left(\ln\;t-\ln\;u\right)}{\left(\ln\;t-\ln\;u\right)}^{p-1}\frac{z\left(u\right)}{u}\text{d}u\\ +\frac{e^{\left(\left(\varrho -1\right)/\varrho \right)\ln\;t}{\left(\ln\;t\right)}^{p+1}}{\varrho^{p}\left(\lambda -l-\varrho^{p}\Gamma \left(p+2\right)\right)}\left(\frac{l-\lambda }{\varrho^{q}\Gamma \left(p+q\right)}\int_{1}^{e}e^{\left(\left(\varrho -1\right)/\varrho \right)\ln\;u}{\left(1-\ln\;u\right)}^{p+q-1}\frac{\xi \left(u,z\left(u\right)\right)}{u}\text{d}u\right.\\ +\frac{1}{\varrho^{q-p}\Gamma \left(q\right)}\int_{1}^{e}e^{\left(\left(\varrho -1\right)/\varrho \right)\ln\;u}{\left(1-\ln\;u\right)}^{q-1}\frac{\xi \left(u,z\left(u\right)\right)}{u}\text{d}u\\ -\frac{\lambda \left(\lambda -l\right)}{\Gamma \left(p\right)}\int_{1}^{e}e^{\left(\left(\varrho -1\right)/\varrho \right)\ln\;u}{\left(1-\ln\;u\right)}^{p-1}\frac{z\left(u\right)}{u}\text{d}u\\ \left.+\frac{l-\lambda +\varrho^{p}\Gamma \left(p+1\right)}{\varrho^{\vartheta }\Gamma \left(p+1\right)\Gamma \left(\vartheta \right)}\int_{1}^{\eta }e^{\left(\left(\varrho -1\right)/\varrho \right)\left(\ln\;\eta -\ln\;u\right)}{\left(\ln\;\eta -\ln\;u\right)}^{\vartheta -1}\frac{z\left(u\right)}{u}\text{d}u\right)\\ +\frac{e^{\left(\left(\varrho -1\right)/\varrho \right)\ln\;t}{\left(\ln\;t\right)}^{p}}{\varrho^{p+\vartheta }\Gamma \left(p+1\right)\Gamma \left(\vartheta \right)}\int_{1}^{\eta }e^{\left(\left(\varrho -1\right)/\varrho \right)\left(\ln\;\eta -\ln\;u\right)}{\left(\ln\;\eta -\ln\;u\right)}^{\vartheta -1}\frac{z\left(u\right)}{u}\text{d}u. \end{array}$$

It should be noted that if the operator *𝔍* has a fixed point, the solution of equation (15) exists.

### 3.1. Existence Result

We now introduce the following conditions to confirm our existence result with the support of fixed point procedure.

**Theorem 2 .**

Let us imagine that the continuous function *ξ* : [1, *e*] × *ℝ*⟶*ℝ* satisfying the following inferences:

- |*ξ*(*t*, *z*(*t*))| ≤ *k*(*t*)∀ (*t*, *z*(*t*)) ∈ [1, *e*] × *ℝ* and *k*(*t*) ∈ (*𝒞*[1, *e*], *ℝ*^+^), with sup_*t*_|*k*(*t*)|=‖*k*‖
- |*ξ*(*t*, *z*(*t*)) − *ξ*(*t*, *z*_1_(*t*))| ≤ *χ*|*z*(*t*) − *z*_1_(*t*)|∀ *t* ∈ [1, *e*], *z*, *z*_1_ ∈ *𝒞*[1, *e*] and *χ* > 0

Then, the FLEs (15) has a minimum of one solution on [1, *e*] if

(28) $$\begin{array}{c} {ϒ}_{3}<1, \end{array}$$

where

(29) $$\begin{array}{c} {ϒ}_{3}=\frac{\left|l-\lambda \right|\chi }{\varrho^{p+q}\left|\lambda -l-\varrho^{p}\Gamma \left(p+2\right)\right|\Gamma \left(p+q+1\right)}+\frac{\chi }{\varrho^{q}\Gamma \left(q+1\right)\left|\lambda -l-\varrho^{p}\Gamma \left(p+2\right)\right|}\\ +\frac{\lambda \left|\lambda -l\right|}{\varrho^{p}\left|\lambda -l-\varrho^{p}\Gamma \left(p+2\right)\right|\Gamma \left(p+1\right)}+\frac{{\left(\text{ln}\eta \right)}^{\vartheta }\left|l-\lambda +\varrho^{p}\Gamma \left(p+1\right)\right|}{\varrho^{p+\vartheta }\Gamma \left(p+1\right)\Gamma \left(\vartheta +1\right)\left|\lambda -l-\varrho^{p}\Gamma \left(p+2\right)\right|}+\frac{{\left(\ln\;\eta \right)}^{\vartheta }}{\varrho^{p+\vartheta }\Gamma \left(p+1\right)\Gamma \left(\vartheta +1\right)}. \end{array}$$

**Proof**

Choose *𝔯*=(ϒ_1_‖*k*‖/(1 − ϒ_2_)), where

(30) $$\begin{array}{c} {ϒ}_{1}=\left\{\frac{1}{\varrho^{p+q}\Gamma \left(p+q+1\right)}+\frac{\left|l-\lambda \right|}{\varrho^{p+q}\left|\lambda -l-\varrho^{p}\Gamma \left(p+2\right)\right|\Gamma \left(p+q+1\right)}+\frac{1}{\varrho^{q}\Gamma \left(q+1\right)\left|\lambda -l-\varrho^{p}\Gamma \left(p+2\right)\right|}\right\},\\ {ϒ}_{2}=\left\{\frac{\lambda }{\varrho^{p}\Gamma \left(p+1\right)}+\frac{\lambda \left|\lambda -l\right|}{\varrho^{p}\left|\lambda -l-\varrho^{p}\Gamma \left(p+2\right)\right|\Gamma \left(p+1\right)}+\frac{{\left(\text{ln}\eta \right)}^{\vartheta }\left|l-\lambda +\varrho^{p}\Gamma \left(p+1\right)\right|}{\varrho^{p+\vartheta }\Gamma \left(p+1\right)\Gamma \left(\vartheta +1\right)\left|\lambda -l-\varrho^{p}\Gamma \left(p+2\right)\right|}\right.\\ \left.+\frac{{\left(\ln\;\eta \right)}^{\vartheta }}{\varrho^{p+\vartheta }\Gamma \left(p+1\right)\Gamma \left(\vartheta +1\right)}\right\}. \end{array}$$

Define the closed ball ${\bar{B}}_{𝔯}=\left\{w\in 𝒞\left[1,e\right]:\left\|w\right\|\leq 𝔯\right\}$.

By combining the operators ${𝔍}_{1},{𝔍}_{2}\in {\bar{B}}_{𝔯}$ which are described as follows:

(31) $$\begin{array}{c} J_{1}z\left(t\right)=\frac{1}{\varrho^{p+q}\Gamma \left(p+q\right)}\int_{1}^{t}e^{\left(\left(\varrho -1\right)/\varrho \right)\left(\ln\;t-\ln\;u\right)}{\left(\ln\;t-\ln\;u\right)}^{p+q-1}\frac{\xi \left(u,z\left(u\right)\right)}{u}\text{d}u\\ -\frac{\lambda }{\varrho^{p}\Gamma \left(p\right)}\int_{1}^{t}e^{\left(\left(\varrho -1\right)/\varrho \right)\left(\ln\;t-\ln\;u\right)}{\left(\ln\;t-\ln\;u\right)}^{p-1}\frac{z\left(u\right)}{u}\text{d}u,\\ J_{2}z\left(t\right)=\frac{e^{\left(\left(\varrho -1\right)/\varrho \right)\ln\;t}{\left(\ln\;t\right)}^{p+1}}{\varrho^{p}\left(\lambda -l-\varrho^{p}\Gamma \left(p+2\right)\right)}\left(\frac{l-\lambda }{\varrho^{q}\Gamma \left(p+q\right)}\int_{1}^{e}e^{\left(\left(\varrho -1\right)/\varrho \right)\ln\;u}{\left(1-\ln\;u\right)}^{p+q-1}\frac{\xi \left(u,z\left(u\right)\right)}{u}\text{d}u\right.\\ +\frac{1}{\varrho^{q-p}\Gamma \left(q\right)}\int_{1}^{e}e^{\left(\left(\varrho -1\right)/\varrho \right)\ln\;u}{\left(1-\ln\;u\right)}^{q-1}\frac{\xi \left(u,z\left(u\right)\right)}{u}\text{d}u-\frac{\lambda \left(\lambda -l\right)}{\Gamma \left(p\right)}\int_{1}^{e}e^{\left(\left(\varrho -1\right)/\varrho \right)\ln\;u}{\left(1-\ln\;u\right)}^{p-1}\frac{z\left(u\right)}{u}\text{d}u\\ \left.+\frac{l-\lambda +\varrho^{p}\Gamma \left(p+1\right)}{\varrho^{\vartheta }\Gamma \left(p+1\right)\Gamma \left(\vartheta \right)}\int_{1}^{\eta }e^{\left(\left(\varrho -1\right)/\varrho \right)\left(\ln\;\eta -\ln\;u\right)}{\left(\ln\;\eta -\ln\;u\right)}^{\vartheta -1}\frac{z\left(u\right)}{u}\text{d}u\right)\\ +\frac{e^{\left(\left(\varrho -1\right)/\varrho \right)\ln\;t}{\left(\ln\;t\right)}^{p}}{\varrho^{p+\vartheta }\Gamma \left(p+1\right)\Gamma \left(\vartheta \right)}\int_{1}^{\eta }e^{\left(\left(\varrho -1\right)/\varrho \right)\left(\ln\;\eta -\ln\;u\right)}{\left(\ln\;\eta -\ln\;u\right)}^{\vartheta -1}\frac{z\left(u\right)}{u}\text{d}u. \end{array}$$

Keep this in mind $𝔍z\left(t\right)={𝔍}_{1}z\left(t\right)+{𝔍}_{2}z\left(t\right)\in {\bar{B}}_{𝔯}$ on [1, *e*].

First, we will show that $𝔍z\left(t\right)={𝔍}_{1}z_{1}\left(t\right)+{𝔍}_{2}z_{2}\left(t\right)\in {\bar{B}}_{𝔯}$, for each $z_{1},z_{2}\in {\bar{B}}_{𝔯}$.

(32) $$\begin{array}{c} \left\|J_{1}z_{1}+J_{2}z_{2}\right\|=\underset{t\in \left[1,e\right]}{\text{sup}}\left|J_{1}z_{1}\left(t\right)+J_{2}z_{2}\left(t\right)\right|\\ =\underset{t\in \left[1,e\right]}{\text{sup}}\left\{|\frac{1}{\varrho^{p+q}\Gamma \left(p+q\right)}\int_{1}^{t}e^{\left(\left(\varrho -1\right)/\varrho \right)\left(\ln\;t-\ln\;u\right)}{\left(\ln\;t-\ln\;u\right)}^{p+q-1}\frac{\xi \left(u,z\left(u\right)\right)}{u}\text{d}u\right.\\ -\frac{\lambda }{\varrho^{p}\Gamma \left(p\right)}\int_{1}^{t}e^{\left(\left(\varrho -1\right)/\varrho \right)\left(\ln\;t-\ln\;u\right)}{\left(\ln\;t-\ln\;u\right)}^{p-1}\frac{z\left(u\right)}{u}\text{d}u\\ +\frac{e^{\left(\left(\varrho -1\right)/\varrho \right)\ln\;t}{\left(\ln\;t\right)}^{p+1}}{\varrho^{p}\left(\lambda -l-\varrho^{p}\Gamma \left(p+2\right)\right)}\left(\frac{l-\lambda }{\varrho^{q}\Gamma \left(p+q\right)}\int_{1}^{e}e^{\left(\left(\varrho -1\right)/\varrho \right)\ln\;u}{\left(1-\ln\;u\right)}^{p+q-1}\frac{\xi \left(u,z\left(u\right)\right)}{u}\text{d}u\right.\\ +\frac{1}{\varrho^{q-p}\Gamma \left(q\right)}\int_{1}^{e}e^{\left(\left(\varrho -1\right)/\varrho \right)\ln\;u}{\left(1-\ln\;u\right)}^{q-1}\frac{\xi \left(u,z\left(u\right)\right)}{u}\text{d}u-\frac{\lambda \left(\lambda -l\right)}{\Gamma \left(p\right)}\int_{1}^{e}e^{\left(\left(\varrho -1\right)/\varrho \right)\ln\;u}{\left(1-\ln\;u\right)}^{p-1}\frac{z\left(u\right)}{u}\text{d}u\\ \left.+\frac{l-\lambda +\varrho^{p}\Gamma \left(p+1\right)}{\varrho^{\vartheta }\Gamma \left(p+1\right)\Gamma \left(\vartheta \right)}\int_{1}^{\eta }e^{\left(\left(\varrho -1\right)/\varrho \right)\left(\ln\;\eta -\ln\;u\right)}{\left(\ln\;\eta -\ln\;u\right)}^{\vartheta -1}\frac{z\left(u\right)}{u}\text{d}u\right)\\ \left.+\frac{e^{\left(\left(\varrho -1\right)/\varrho \right)\ln\;t}{\left(\ln\;t\right)}^{p}}{\varrho^{p+\vartheta }\Gamma \left(p+1\right)\Gamma \left(\vartheta \right)}\int_{1}^{\eta }e^{\left(\left(\varrho -1\right)/\varrho \right)\left(\ln\;\eta -\ln\;u\right)}{\left(\ln\;\eta -\ln\;u\right)}^{\vartheta -1}\frac{z\left(u\right)}{u}\text{d}u|\right\}\\ \leq \underset{t\in \left[1,e\right]}{\text{sup}}\left\{\frac{1}{\varrho^{p+q}\Gamma \left(p+q\right)}\int_{1}^{t}e^{\left(\left(\varrho -1\right)/\varrho \right)\left(\ln\;t-\ln\;u\right)}{\left(\ln\;t-\ln\;u\right)}^{p+q-1}\frac{\left|\xi \left(u,z\left(u\right)\right)\right|}{u}\text{d}u\right.\\ -\frac{\lambda }{\varrho^{p}\Gamma \left(p\right)}\int_{1}^{t}e^{\left(\left(\varrho -1\right)/\varrho \right)\left(\ln\;t-\ln\;u\right)}{\left(\ln\;t-\ln\;u\right)}^{p-1}\frac{\left|z\left(u\right)\right|}{u}\text{d}u\\ +\frac{e^{\left(\left(\varrho -1\right)/\varrho \right)\ln\;t}{\left(\ln\;t\right)}^{p+1}}{\varrho^{p}\left(\lambda -l-\varrho^{p}\Gamma \left(p+2\right)\right)}\left(\frac{l-\lambda }{\varrho^{q}\Gamma \left(p+q\right)}\int_{1}^{e}e^{\left(\left(\varrho -1\right)/\varrho \right)\ln\;u}{\left(1-\ln\;u\right)}^{p+q-1}\frac{\left|\xi \left(u,z\left(u\right)\right)\right|}{u}\text{d}u\right.\\ +\frac{1}{\varrho^{q-p}\Gamma \left(q\right)}\int_{1}^{e}e^{\left(\left(\varrho -1\right)/\varrho \right)\ln\;u}{\left(1-\ln\;u\right)}^{q-1}\frac{\left|\xi \left(u,z\left(u\right)\right)\right|}{u}\text{d}u-\frac{\lambda \left(\lambda -l\right)}{\Gamma \left(p\right)}\int_{1}^{e}e^{\left(\left(\varrho -1\right)/\varrho \right)\ln\;u}{\left(1-\ln\;u\right)}^{p-1}\frac{\left|z\left(u\right)\right|}{u}\text{d}u\\ +\frac{l-\lambda +\varrho^{p}\Gamma \left(p+1\right)}{\varrho^{\vartheta }\Gamma \left(p+1\right)\Gamma \left(\vartheta \right)}\int_{1}^{\eta }e^{\left(\left(\varrho -1\right)/\varrho \right)\left(\ln\;\eta -\ln\;u\right)}{\left(\ln\;\eta -\ln\;u\right)}^{\vartheta -1}\frac{\left|z\left(u\right)\right|}{u}\text{d}u\\ \left.+\frac{e^{\left(\left(\varrho -1\right)/\varrho \right)\ln\;t}{\left(\ln\;t\right)}^{p}}{\varrho^{p+\vartheta }\Gamma \left(p+1\right)\Gamma \left(\vartheta \right)}\int_{1}^{\eta }e^{\left(\left(\varrho -1\right)/\varrho \right)\left(\ln\;\eta -\ln\;u\right)}{\left(\ln\;\eta -\ln\;u\right)}^{\vartheta -1}\frac{\left|z\left(u\right)\right|}{u}\text{d}u\right\}. \end{array}$$

Taking advantage of the *e*^((ϱ − 1)/ϱ)ln *t*^ ≤ *e*^((ϱ − 1)/ϱ)(ln *t* − ln *u*)^ ≤ 1, for 0 ≤  ln *u* <  ln *η* <  ln *t* ≤ 1, and from *I*_1_, we get

(33) $$\begin{array}{c} \left\|J_{1}z_{1}+J_{2}z_{2}\right\|\leq \left\|k\right\|\underset{t\in \left[1,e\right]}{\text{sup}}\left\{\frac{1}{\varrho^{p+q}\Gamma \left(p+q\right)}\int_{1}^{t}{\left(\ln\;t-\ln\;u\right)}^{p+q-1}\frac{\text{d}u}{u}\right.\\ +\frac{\left|l-\lambda \right|{\left(\text{ln}t\right)}^{p+1}}{\varrho^{p+q}\left|\lambda -l-\varrho^{p}\Gamma \left(p+2\right)\right|\Gamma \left(p+1\right)}\int_{1}^{e}{\left(1-\ln\;u\right)}^{p+q-1}\frac{\text{d}u}{u}\\ \left.+\frac{{\left(\text{ln}t\right)}^{p+1}}{\varrho^{q}\Gamma \left(q\right)\left|\lambda -l-\varrho^{p}\Gamma \left(p+2\right)\right|}\int_{1}^{e}{\left(1-\ln\;u\right)}^{q-1}\frac{\text{d}u}{u}\right\}+\frac{\left\|z_{1}\right\|\lambda }{\varrho^{p}\Gamma \left(p\right)}\underset{t\in \left[1,e\right]}{\text{sup}}\left(\int_{1}^{t}{\left(\ln\;t-\ln\;u\right)}^{p-1}\frac{\text{d}u}{u}\right)\\ +\left\|z_{2}\right\|\underset{t\in \left[1,e\right]}{\text{sup}}\left\{\frac{\lambda \left|\lambda -l\right|{\left(\ln\;t\right)}^{p+1}}{\varrho^{p}\left|\lambda -l-\varrho^{p}\Gamma \left(p+2\right)\right|\Gamma \left(p\right)}\int_{1}^{e}{\left(1-\ln\;u\right)}^{p-1}\frac{\text{d}u}{u}\right.\\ \left.+\left(\frac{{\left(\text{ln}\eta \right)}^{\vartheta }\left|l-\lambda +\varrho^{p}\Gamma \left(p+1\right)\right|}{\varrho^{p+\vartheta }\Gamma \left(p+1\right)\Gamma \left(\vartheta +1\right)\left|\lambda -l-\varrho^{p}\Gamma \left(p+2\right)\right|}+\frac{{\left(\ln\;t\right)}^{p}}{\varrho^{p+\vartheta }\Gamma \left(p+1\right)\Gamma \left(\vartheta \right)}\right)\int_{1}^{\eta }{\left(\ln\;\eta -\ln\;u\right)}^{\vartheta -1}\frac{\text{d}u}{u}\right\}\\ \leq \left\|k\right\|\left\{\frac{1}{\varrho^{p+q}\Gamma \left(p+q+1\right)}+\frac{\left|l-\lambda \right|}{\varrho^{p+q}\left|\lambda -l-\varrho^{p}\Gamma \left(p+2\right)\right|\Gamma \left(p+q+1\right)}+\frac{1}{\varrho^{q}\Gamma \left(q+1\right)\left|\lambda -l-\varrho^{p}\Gamma \left(p+2\right)\right|}\right\}\\ +r\left\{\frac{\lambda }{\varrho^{p}\Gamma \left(p+1\right)}+\frac{\lambda \left|\lambda -l\right|}{\varrho^{p}\left|\lambda -l-\varrho^{p}\Gamma \left(p+2\right)\right|\Gamma \left(p+1\right)}+\frac{{\left(\text{ln}\eta \right)}^{\vartheta }\left|l-\lambda +\varrho^{p}\Gamma \left(p+1\right)\right|}{\varrho^{p+\vartheta }\Gamma \left(p+1\right)\Gamma \left(\vartheta +1\right)\left|\lambda -l-\varrho^{p}\Gamma \left(p+2\right)\right|}\right.\\ \left.+\frac{{\left(\ln\;\eta \right)}^{\vartheta }}{\varrho^{p+\vartheta }\Gamma \left(p+1\right)\Gamma \left(\vartheta +1\right)}\right\}\leq \left\|k\right\|{ϒ}_{1}+r{ϒ}_{2}\leq r. \end{array}$$

Hence, $𝔍z\left(t\right)={𝔍}_{1}z_{1}\left(t\right)+{𝔍}_{2}z_{2}\left(t\right)\in {\bar{B}}_{𝔯}$.

Second, we will demonstrate that *𝔍*_2_ is a contraction operator. Let *α*_1_ and *α*_2_ be two elements in Banach space (*𝒞*[1, *e*], ‖.‖) such that

(34) $$\begin{array}{c} \left\|J_{2}\alpha_{1}-J_{2}\alpha_{2}\right\|=\underset{t\in \left[1,e\right]}{\text{sup}}\left|J_{1}\alpha_{1}\left(t\right)-J_{2}\alpha_{2}\left(t\right)\right|\\ \leq \underset{t\in \left[1,e\right]}{\text{sup}}\left\{\frac{e^{\left(\left(\varrho -1\right)/\varrho \right)\ln\;t}{\left(\ln\;t\right)}^{p+1}}{\varrho^{p}\left|\lambda -l-\varrho^{p}\Gamma \left(p+2\right)\right|}\left[\frac{\left|l-\lambda \right|}{\varrho^{q}\Gamma \left(p+q\right)}\int_{1}^{e}e^{\left(\left(\varrho -1\right)/\varrho \right)\ln\;u}{\left(1-\ln\;u\right)}^{p+q-1}\frac{\left|\xi \left(u,\alpha_{1}\left(u\right)\right)-\xi \left(u,\alpha_{2}\left(u\right)\right)\right|}{u}\text{d}u\right.\right.\\ +\frac{1}{\varrho^{q-p}\Gamma \left(q\right)}\int_{1}^{e}e^{\left(\left(\varrho -1\right)/\varrho \right)\ln\;u}{\left(1-\ln\;u\right)}^{q-1}\frac{\left|\xi \left(u,\alpha_{1}\left(u\right)\right)-\xi \left(u,\alpha_{2}\left(u\right)\right)\right|}{u}\text{d}u\\ +\frac{\lambda \left|\lambda -l\right|}{\Gamma \left(p\right)}\int_{1}^{e}e^{\left(\left(\varrho -1\right)/\varrho \right)\ln\;u}{\left(1-\ln\;u\right)}^{p-1}\frac{\left|\alpha_{1}\left(u\right)-\alpha_{2}\left(u\right)\right|}{u}\text{d}u\\ +\left.\frac{\left|l-\lambda +\varrho^{p}\Gamma \left(p+1\right)\right|}{\varrho^{\vartheta }\Gamma \left(p+1\right)\Gamma \left(\vartheta \right)}\int_{1}^{\eta }e^{\left(\left(\varrho -1\right)/\varrho \right)\left(\ln\;\eta -\ln\;u\right)}{\left(\ln\;\eta -\ln\;u\right)}^{\vartheta -1}\frac{\left|\alpha_{1}\left(u\right)-\alpha_{2}\left(u\right)\right|}{u}\text{d}u\right]\\ \;\left.+\frac{e^{\left(\left(\varrho -1\right)/\varrho \right)\ln\;t}{\left(\text{ln}t\right)}^{p}}{\varrho^{p+\vartheta }\Gamma \left(p+1\right)\Gamma \left(\vartheta \right)}\int_{1}^{\eta }e^{\left(\left(\varrho -1\right)/\varrho \right)\left(\ln\;\eta -\ln\;u\right)}{\left(\ln\;\eta -\ln\;u\right)}^{\vartheta -1}\frac{\left|\alpha_{1}\left(u\right)-\alpha_{2}\left(u\right)\right|}{u}\text{d}u\right\}. \end{array}$$

Taking advantage of the *e*^((ϱ − 1)/ϱ)ln *t*^ ≤ *e*^((ϱ − 1)/ϱ)(ln *t* − ln *u*)^ ≤ 1 for 0 ≤  ln *u* <  ln *η* <  ln *t* ≤ 1, and from *I*_2_, we get

(35) $$\begin{array}{c} \left\|J_{2}\alpha_{1}-J_{2}\alpha_{2}\right\|\leq \left\{\frac{\left|l-\lambda \right|\chi }{\varrho^{p+q}\left|\lambda -l-\varrho^{p}\Gamma \left(p+2\right)\right|\Gamma \left(p+q+1\right)}+\frac{\chi }{\varrho^{q}\Gamma \left(q+1\right)\left|\lambda -l-\varrho^{p}\Gamma \left(p+2\right)\right|}\right.\\ +\frac{\lambda \left|\lambda -l\right|}{\varrho^{p}\left|\lambda -l-\varrho^{p}\Gamma \left(p+2\right)\right|\Gamma \left(p+1\right)}+\frac{{\left(\text{ln}\eta \right)}^{\vartheta }\left|l-\lambda +\varrho^{p}\Gamma \left(p+1\right)\right|}{\varrho^{p+\vartheta }\Gamma \left(p+1\right)\Gamma \left(\vartheta +1\right)\left|\lambda -l-\varrho^{p}\Gamma \left(p+2\right)\right|}\\ \left.+\frac{{\left(\ln\;\eta \right)}^{\vartheta }}{\varrho^{p+\vartheta }\Gamma \left(p+1\right)\Gamma \left(\vartheta +1\right)}\right\}\left\|\alpha_{1}-\alpha_{2}\right\|\leq {ϒ}_{3}\left\|\alpha_{1}-\alpha_{2}\right\|. \end{array}$$

Hence, *𝔍*_2_ is a contraction map, as implied by assumption that ϒ_3_ < 1.

Finally, we shall demonstrate that *𝔍*_1_ is continuous and compact.

To begin with, since *ξ* is a continuous function on *t* ∈ [1, *e*], operator *𝔍*_1_ is also continuous.

*𝔍*_1_ must be uniformly bounded and equicontinuous on ${\bar{B}}_{𝔯}$ in order to be seen to be compact.

(36) $$\begin{array}{c} \left\|J_{1}z\right\|=\underset{t\in \left[1,e\right]}{\text{sup}}\left|J_{1}z\left(t\right)\right|\\ \leq \underset{t\in \left[1,e\right]}{\text{sup}}\left\{\frac{1}{\varrho^{p+q}\Gamma \left(p+q\right)}\int_{1}^{t}e^{\left(\left(\varrho -1\right)/\varrho \right)\left(\ln\;\eta -\ln\;u\right)}{\left(\ln\;\eta -\ln\;u\right)}^{p+q-1}\frac{\left|\xi \left(u,z\left(u\right)\right)\right|}{u}\text{d}u\right.\\ \left.+\frac{\left|\lambda \right|}{\varrho^{p}\Gamma \left(p\right)}\int_{1}^{t}e^{\left(\left(\varrho -1\right)/\varrho \right)\left(\ln\;\eta -\ln\;u\right)}{\left(\ln\;\eta -\ln\;u\right)}^{p-1}\frac{\left|z\left(u\right)\right|}{u}\text{d}u\right\}\\ \leq \left\|k\right\|\underset{t\in \left[1,e\right]}{\text{sup}}\left\{\frac{1}{\varrho^{p+q}\Gamma \left(p+q\right)}\int_{1}^{t}{\left(\ln\;t-\ln\;u\right)}^{p+q-1}\frac{\text{d}u}{u}\right\}+r\underset{t\in \left[1,e\right]}{\text{sup}}\left\{\frac{\left|\lambda \right|}{\varrho^{p}\Gamma \left(p\right)}\int_{1}^{t}{\left(\ln\;\eta -\ln\;u\right)}^{p-1}\frac{\text{d}u}{u}\right\}\\ \leq \frac{\left|k\right|}{\varrho^{p+q}\Gamma \left(p+q+1\right)}+\frac{\left|\lambda \right|r}{\varrho^{p}\Gamma \left(p\right)}<\infty . \end{array}$$

This proves that *𝔍*_1_ is uniformly bounded.

The compactness of operator *𝔍*_1_ is then demonstrated. For each 1 < *t*_1_ < *t*_2_ < *e*, we get

(37) $$\begin{array}{c} \left|J_{1}z\left(t_{1}\right)-J_{1}z\left(t_{2}\right)\right|=|\frac{1}{\varrho^{p+q}\Gamma \left(p+q\right)}\int_{1}^{t_{2}}e^{\left(\left(\varrho -1\right)/\varrho \right)\left(\ln\;t_{2}-\ln\;u\right)}{\left(\ln\;t_{2}-\ln\;u\right)}^{p+q-1}\frac{\xi \left(u,z\left(u\right)\right)}{u}\text{d}u\\ -\frac{\left|\lambda \right|}{\varrho^{p}\Gamma \left(p\right)}\int_{1}^{t_{2}}e^{\left(\left(\varrho -1\right)/\varrho \right)\left(\ln\;t_{2}-\ln\;u\right)}{\left(\ln\;t_{2}-\ln\;u\right)}^{p-1}\frac{z\left(u\right)}{u}\text{d}u\\ -\frac{1}{\varrho^{p+q}\Gamma \left(p+q\right)}\int_{1}^{t_{1}}e^{\left(\left(\varrho -1\right)/\varrho \right)\left(\ln\;t_{1}-\ln\;u\right)}{\left(\ln\;t_{1}-\ln\;u\right)}^{p+q-1}\frac{\xi \left(u,z\left(u\right)\right)}{u}\text{d}u\\ +\frac{\left|\lambda \right|}{\varrho^{p}\Gamma \left(p\right)}\int_{1}^{t_{1}}e^{\left(\left(\varrho -1\right)/\varrho \right)\left(\ln\;t_{1}-\ln\;u\right)}{\left(\ln\;t_{1}-\ln\;u\right)}^{p-1}\frac{z\left(u\right)}{u}\text{d}u|,\\ \left|J_{1}z\left(t_{1}\right)-J_{1}z\left(t_{2}\right)\right|=|\frac{1}{\varrho^{p+q}\Gamma \left(p+q\right)}\int_{1}^{t_{1}}\left[e^{\left(\left(\varrho -1\right)/\varrho \right)\ln\left(t_{2}/u\right)}{\left(\ln\frac{t_{2}}{u}\right)}^{p+q-1}-e^{\left(\left(\varrho -1\right)/\varrho \right)\ln\left(t_{1}/u\right)}{\left(\ln\frac{t_{1}}{u}\right)}^{p+q-1}\right]\frac{\xi \left(u,z\left(u\right)\right)}{u}\text{d}u\\ +\frac{1}{\varrho^{p+q}\Gamma \left(p+q\right)}\int_{t_{1}}^{t_{2}}e^{\left(\left(\varrho -1\right)/\varrho \right)\ln\left(t_{2}/u\right)}{\left(\text{ln}\frac{t_{2}}{u}\right)}^{p+q-1}\frac{\xi \left(u,z\left(u\right)\right)}{u}\text{d}u\\ -\frac{\lambda }{\varrho^{p}\Gamma \left(p\right)}\int_{1}^{t_{1}}\left[e^{\left(\left(\varrho -1\right)/\varrho \right)\ln\left(t_{2}/u\right)}{\left(\ln\frac{t_{2}}{u}\right)}^{p-1}-e^{\left(\left(\varrho -1\right)/\varrho \right)\ln\left(t_{1}/u\right)}{\left(\text{ln}\frac{t_{1}}{u}\right)}^{p-1}\right]\frac{z\left(u\right)}{u}\text{d}u\\ +\frac{\lambda }{\varrho^{p}\Gamma \left(p\right)}\int_{t_{1}}^{t_{2}}e^{\left(\left(\varrho -1\right)/\varrho \right)\left(\ln\;t_{2}-\ln\;u\right)}{\left(\ln\;t_{2}-\ln\;u\right)}^{p-1}\frac{z\left(u\right)}{u}\text{d}u|\\ \leq \frac{\left\|k\right\|}{\varrho^{p+q}\Gamma \left(p+q\right)}\left\{\int_{1}^{t_{1}}e^{\left(\left(\varrho -1\right)/\varrho \right)\ln\left(t_{1}/u\right)}\left|e^{\left(\left(\varrho -1\right)/\varrho \right)\ln\left(t_{2}/t_{1}\right)}{\left(\text{ln}\frac{t_{2}}{u}\right)}^{p+q-1}-{\left(\text{ln}\frac{t_{1}}{u}\right)}^{p+q-1}\right|\frac{du}{u}\right.\\ \left.+\int_{t_{1}}^{t_{2}}e^{\left(\left(\varrho -1\right)/\varrho \right)\ln\left(t_{2}/u\right)}{\left|\text{ln}\frac{t_{2}}{u}\right|}^{p+q-1}\frac{\text{d}u}{u}\right\}\\ +\frac{\lambda r}{\varrho^{p}\Gamma \left(p\right)}\left\{\int_{1}^{t_{1}}e^{\left(\left(\varrho -1\right)/\varrho \right)\ln\left(t_{1}/u\right)}\left|e^{\left(\left(\varrho -1\right)/\varrho \right)\ln\left(t_{2}/u\right)}{\left(\text{ln}\frac{t_{2}}{u}\right)}^{p-1}-{\left(\text{ln}\frac{t_{1}}{u}\right)}^{p-1}\right|\frac{\text{d}u}{u}\right.\\ \left.+\int_{t_{1}}^{t_{2}}e^{\left(\left(\varrho -1\right)/\varrho \right)\left(\ln\;t_{2}-\ln\;u\right)}{\left|\ln\;t_{2}-\ln\;u\right|}^{p-1}\frac{\text{d}u}{u}\right\}. \end{array}$$

Taking advantage of the *e*^((ϱ − 1)/ϱ)ln(*t*_2_/*u*)^ < *e*^((ϱ − 1)/ϱ)(ln(*t*_1_/*u*))^ ≤ 1, *e*^((ϱ − 1)/ϱ)(ln(*t*_2_/*t*_1_))^ ≤ 1, for each 1 ≤ *u* < *t*_1_ < *t*_2_ ≤ *e*, and from the first mean value theorem, we get

(38) $$\begin{array}{c} \left|J_{1}z\left(t_{1}\right)-J_{1}z\left(t_{2}\right)\right|\leq \frac{\left\|k\right\|}{\varrho^{p+q}\Gamma \left(p+q\right)}\left\{\int_{1}^{t_{1}}\left|{\left(\text{ln}\frac{t_{2}}{u}\right)}^{p+q-1}-{\left(\text{ln}\frac{t_{1}}{u}\right)}^{p+q-1}\right|\frac{\text{d}u}{u}+\int_{t_{1}}^{t_{2}}{\left|\text{ln}\frac{t_{2}}{u}\right|}^{p+q-1}\frac{\text{d}u}{u}\right\}\\ +\frac{\lambda r}{\varrho^{p}\Gamma \left(p\right)}\left\{\int_{1}^{t_{1}}\left|{\left(\ln\frac{t_{2}}{u}\right)}^{p-1}-{\left(\text{ln}\frac{t_{1}}{u}\right)}^{p-1}\right|\frac{\text{d}u}{u}+\int_{t_{1}}^{t_{2}}{\left|\ln\;t_{2}-\ln\;u\right|}^{p-1}\frac{\text{d}u}{u}\right\}. \end{array}$$

Taking the limit as *t*_2_⟶*t*_1_, we have |*𝔍*_1_*z*(*t*_1_) − *𝔍*_1_*z*(*t*_2_)|⟶0.

*𝔍*_1_ is an equicontinuous as a result of this. We may conclude that *𝔍*_1_ compacts on ${\bar{B}}_{𝔯}$ based on the Arzela Ascoli theorem. As a result, in ${\bar{B}}_{𝔯}$, there is a point *z* so that *z*=*𝔍*_1_*z*. As a consequence, equation (1) has at least one solution on [1, *e*]. □

### 3.2. Uniqueness Result

**Theorem 3 .**

Let us imagine that the continuous function *ξ* : [1, *e*] × *ℝ*⟶*ℝ* satisfying the inferences (I), (II) and

  (I3) *ℵ*=sup_*t*∈[1, *e*]_|*ξ*(*t*, 0)| < *∞*

If

(39) $$\begin{array}{c} \Omega =\chi {ϒ}_{1}+{ϒ}_{2}\leq 1, \end{array}$$

then the FLEs (15) have a unique solution on [1, *e*].

**Proof**

Choose *𝔯*_1_ > (ϒ_1_*ℵ*/(1 − ϒ_1_ − ϒ_2_)), to define the closed ball ${\bar{B}}_{{𝔯}_{1}}=\left\{w\in 𝒞\left[1,e\right]:\left\|w\right\|\leq {𝔯}_{1}\right\}$.

$𝔍{\bar{B}}_{{𝔯}_{1}}$ must be bounded. So, we must demonstrate that $𝔍{\bar{B}}_{{𝔯}_{1}}\subseteq {\bar{B}}_{{𝔯}_{1}}$ and *𝔍* is contractive.

For each *u* ∈ [1, *e*] and $w\in {\bar{B}}_{{𝔯}_{1}}$, we get

(40) $$\begin{array}{c} \left|\xi \left(u,w\left(u\right)\right)\right|=\left|\xi \left(u,w\left(u\right)\right)-\xi \left(u,0\right)+\xi \left(u,0\right)\right|\leq \left|\xi \left(u,w\left(u\right)\right)-\xi \left(u,0\right)\right|+\left|\xi \left(u,0\right)\right|\\ \leq \chi \left|w\left(u\right)\right|+ℵ\leq \chi r_{1}+ℵ. \end{array}$$

Therefore,

(41) $$\begin{array}{c} \left|Jw\left(t\right)\right|\leq \underset{t\in \left[1,e\right]}{\sup}\left\{\frac{1}{\varrho^{p+q}\Gamma \left(p+q\right)}\int_{1}^{t}e^{\left(\left(\varrho -1\right)/\varrho \right)\left(\ln\;t-\ln\;u\right)}{\left(\ln\;t-\ln\;u\right)}^{p+q-1}\frac{\left|\xi \left(u,w\left(u\right)\right)\right|}{u}\text{d}u\right.\\ +\frac{\left|\lambda \right|}{\varrho^{p}\Gamma \left(p\right)}\int_{1}^{t}e^{\left(\left(\varrho -1\right)/\varrho \right)\left(\ln\;t-\ln\;u\right)}{\left(\ln\;t-\ln\;u\right)}^{p-1}\frac{\left|w\left(u\right)\right|}{u}\text{d}u\\ +\frac{e^{\left(\left(\varrho -1\right)/\varrho \right)\ln\;t}{\left(\ln\;t\right)}^{p+1}}{\varrho^{p}\left(\lambda -l-\varrho^{p}\Gamma \left(p+2\right)\right)}\left(\frac{l-\lambda }{\varrho^{q}\Gamma \left(p+q\right)}\int_{1}^{e}e^{\left(\left(\varrho -1\right)/\varrho \right)\ln\;u}{\left(1-\ln\;u\right)}^{p+q-1}\frac{\xi \left(u,w\left(u\right)\right)}{u}\text{d}u\right.\\ +\frac{1}{\varrho^{q-p}\Gamma \left(q\right)}\int_{1}^{e}e^{\left(\left(\varrho -1\right)/\varrho \right)\ln\;u}{\left(1-\ln\;u\right)}^{q-1}\frac{\left|\xi \left(u,w\left(u\right)\right)\right|}{u}\text{d}u\\ +\frac{\left|\lambda \right|\left|\lambda -l\right|}{\Gamma \left(p\right)}\int_{1}^{e}e^{\left(\left(\varrho -1\right)/\varrho \right)\ln\;u}{\left(1-\ln\;u\right)}^{p-1}\frac{\left|w\left(u\right)\right|}{u}\text{d}u\\ \left.+\frac{\left|l-\lambda +\varrho^{p}\Gamma \left(p+1\right)\right|}{\varrho^{\vartheta }\Gamma \left(p+1\right)\Gamma \left(\vartheta \right)}\int_{1}^{\eta }e^{\left(\left(\varrho -1\right)/\varrho \right)\left(\ln\;\eta -\ln\;u\right)}{\left(\ln\;\eta -\ln\;u\right)}^{\vartheta -1}\frac{\left|w\left(u\right)\right|}{u}\text{d}u\right)\\ \left.+\frac{e^{\left(\left(\varrho -1\right)/\varrho \right)\ln\;t}{\left(\ln\;t\right)}^{p}}{\varrho^{p+\vartheta }\Gamma \left(p+1\right)\Gamma \left(\vartheta \right)}\int_{1}^{\eta }e^{\left(\left(\varrho -1\right)/\varrho \right)\left(\ln\;\eta -\ln\;u\right)}{\left(\ln\;\eta -\ln\;u\right)}^{\vartheta -1}\frac{\left|w\left(u\right)\right|}{u}\text{d}u|\right\}\\ \leq \left(\chi r_{1}+ℵ\right)\underset{t\in \left[1,e\right]}{\sup}\left\{\frac{1}{\varrho^{p+q}\Gamma \left(p+q\right)}\int_{1}^{t}e^{\left(\left(\varrho -1\right)/\varrho \right)\left(\ln\;t-\ln\;u\right)}{\left(\ln\;t-\ln\;u\right)}^{p+q-1}\frac{\text{d}u}{u}\right.\\ +\frac{e^{\left(\left(\varrho -1\right)/\varrho \right)\ln\;t}{\left|\ln\;t\right|}^{p+1}}{\varrho^{p}\left|\lambda -l-\varrho^{p}\Gamma \left(p+2\right)\right|}\frac{\left|l-\lambda \right|}{\varrho^{q}\Gamma \left(p+q\right)}\int_{1}^{e}e^{\left(\left(\varrho -1\right)/\varrho \right)\ln\;u}{\left(1-\ln\;u\right)}^{p+q-1}\frac{\text{d}u}{u}\\ \left.+\frac{e^{\left(\left(\varrho -1\right)/\varrho \right)\ln\;t}{\left|\ln\;t\right|}^{p+1}}{\varrho^{p}\left|\lambda -l-\varrho^{p}\Gamma \left(p+2\right)\right|}\frac{1}{\varrho^{q-p}\Gamma \left(q\right)}\int_{1}^{e}e^{\left(\left(\varrho -1\right)/\varrho \right)\ln\;u}{\left(1-\ln\;u\right)}^{q-1}\frac{\text{d}u}{u}\right\}\\ +r_{1}\underset{t\in \left[1,e\right]}{\sup}\left\{\frac{\left|\lambda \right|}{\varrho^{p}\Gamma \left(p\right)}\int_{1}^{t}e^{\left(\left(\varrho -1\right)/\varrho \right)\left(\ln\;t-\ln\;u\right)}{\left(\ln\;t-\ln\;u\right)}^{p-1}\frac{\text{d}u}{u}\right.\\ +\frac{e^{\left(\left(\varrho -1\right)/\varrho \right)\ln\;t}{\left|\ln\;t\right|}^{p+1}}{\varrho^{p}\left|\lambda -l-\varrho^{p}\Gamma \left(p+2\right)\right|}\frac{\left|\lambda \right|\left|\lambda -l\right|}{\Gamma \left(p\right)}\int_{1}^{e}e^{\left(\left(\varrho -1\right)/\varrho \right)\ln\;u}{\left(1-\ln\;u\right)}^{p-1}\frac{\text{d}u}{u}\\ \frac{e^{\left(\left(\varrho -1\right)/\varrho \right)\ln\;t}{\left|\ln\;t\right|}^{p+1}}{\varrho^{p}\left|\lambda -l-\varrho^{p}\Gamma \left(p+2\right)\right|}\frac{\left|l-\lambda +\varrho^{p}\Gamma \left(p+1\right)\right|}{\varrho^{\vartheta }\Gamma \left(p+1\right)\Gamma \left(\vartheta \right)}\int_{1}^{\eta }e^{\left(\left(\varrho -1\right)/\varrho \right)\left(\ln\;\eta -\ln\;u\right)}{\left(\ln\;\eta -\ln\;u\right)}^{\vartheta -1}\frac{\text{d}u}{u}\\ \left.+\frac{e^{\left(\left(\varrho -1\right)/\varrho \right)\ln\;t}|\;\ln\;t{|}^{p}}{\varrho^{p+\vartheta }\Gamma \left(p+1\right)\Gamma \left(\vartheta \right)}\int_{1}^{\eta }e^{\left(\left(\varrho -1\right)/\varrho \right)\left(\ln\;\eta -\ln\;u\right)}{\left(\ln\;\eta -\ln\;u\right)}^{\vartheta -1}\frac{\text{d}u}{u}\right\}. \end{array}$$

Taking advantage of the *e*^((ϱ − 1)/ϱ)ln(*t*_2_/*u*)^ < *e*^((ϱ − 1)/ϱ)(ln(*t*_1_/*u*))^ ≤ 1, *e*^((ϱ − 1)/ϱ)(ln(*t*_2_/*t*_1_))^ ≤ 1, for each 1 ≤ *u* < *t*_1_ < *t*_2_ ≤ *e*,

(42) $$\begin{array}{c} \left|Jw\left(t\right)\right|\leq \left(\chi r_{1}+ℵ\right)\underset{t\in \left[1,e\right]}{\text{sup}}\left\{\frac{1}{\varrho^{p+q}\Gamma \left(p+q\right)}\int_{1}^{t}{\left(\ln\;t-\ln\;u\right)}^{p+q-1}\frac{\text{d}u}{u}\right.\\ +\frac{\left|l-\lambda \right|{\left|\ln\;t\right|}^{p+1}}{\varrho^{p+q}\left|\lambda -l-\varrho^{p}\Gamma \left(p+2\right)\right|\Gamma \left(p+1\right)}\int_{1}^{e}{\left(1-\ln\;u\right)}^{p+q-1}\frac{\text{d}u}{u}\\ \left.+\frac{{\left|\ln\;t\right|}^{p+1}}{\varrho^{q}\Gamma \left(q\right)\left|\lambda -l-\varrho^{p}\Gamma \left(p+2\right)\right|}\int_{1}^{e}{\left(1-\ln\;u\right)}^{q-1}\frac{\text{d}u}{u}\right\}\\ +r_{1}\underset{t\in \left[1,e\right]}{\sup}\left\{\frac{\left|\lambda \right|}{\varrho^{p}\Gamma \left(p\right)}\int_{1}^{t}{\left(\ln\;t-\ln\;u\right)}^{p-1}\frac{\text{d}u}{u}\right.\\ +\frac{\left|\lambda \right|\left|\lambda -l\right|{\left|\ln\;t\right|}^{p+1}}{\varrho^{p}\left|\lambda -l-\varrho^{p}\Gamma \left(p+2\right)\right|\Gamma \left(p\right)}\int_{1}^{e}{\left(1-\ln\;u\right)}^{p-1}\frac{\text{d}u}{u}\\ \frac{\left|l-\lambda +\varrho^{p}\Gamma \left(p+1\right)\right|{\left|\ln\;t\right|}^{p+1}}{\varrho^{\vartheta +p}\Gamma \left(p+1\right)\Gamma \left(\vartheta \right)\left|\lambda -l-\varrho^{p}\Gamma \left(p+2\right)\right|}\int_{1}^{\eta }{\left(\ln\;\eta -\ln\;u\right)}^{\vartheta -1}\frac{\text{d}u}{u}\\ \left.+\frac{{\left|\ln\;t\right|}^{p}}{\varrho^{p+\vartheta }\Gamma \left(p+1\right)\Gamma \left(\vartheta \right)}\int_{1}^{\eta }{\left(\ln\;\eta -\ln\;u\right)}^{\vartheta -1}\frac{\text{d}u}{u}\right\}\\ \leq \left(\chi r_{1}+ℵ\right)\underset{t\in \left[1,e\right]}{\sup}\left\{\frac{1}{\varrho^{p+q}\Gamma \left(p+q+1\right)}+\frac{\left|l-\lambda \right|}{\varrho^{p+q}\left|\lambda -l-\varrho^{p}\Gamma \left(p+2\right)\right|\Gamma \left(p+q+1\right)}\right.\\ \left.+\frac{1}{\varrho^{q}\left|\lambda -l-\varrho^{p}\Gamma \left(p+2\right)\right|\Gamma \left(q+1\right)}\right\}\\ +r_{1}\left\{\frac{\left|\lambda \right|}{\varrho^{p}\Gamma \left(p+1\right)}+\frac{\left|\lambda \right|\left|\lambda -l\right|}{\varrho^{p}\left|\lambda -l-\varrho^{p}\Gamma \left(p+2\right)\right|\Gamma \left(p+1\right)}\right.\\ \left.\frac{\left|l-\lambda +\varrho^{p}\Gamma \left(p+1\right)\right|{\left(\ln\;\eta \right)}^{\vartheta }}{\varrho^{p+\vartheta }\Gamma \left(p+1\right)\Gamma \left(\vartheta +1\right)\left|\lambda -l-\varrho^{p}\Gamma \left(p+2\right)\right|}+\frac{{\left(\ln\;\eta \right)}^{\vartheta }}{\varrho^{p+\vartheta }\Gamma \left(p+1\right)\Gamma \left(\vartheta +1\right)}\right\}\\ \leq \left(\chi r_{1}+ℵ\right){ϒ}_{1}+r_{1}{ϒ}_{2}\leq r_{1}, \end{array}$$

which suggests that ‖*𝔍w*‖ ≤ *𝔯*_1_. Hence, $𝔍{\bar{B}}_{{𝔯}_{1}}\subseteq {\bar{B}}_{{𝔯}_{1}}$.

Now, we will show that *𝔍* is contractive. So, for all *w*_1_, *w*_2_ ∈ *𝒞*[1, *e*], we get

(43) $$\begin{array}{c} \left|Jw_{1}\left(t\right)-Jw_{2}\left(t\right)\right|\leq \underset{t\in \left[1,e\right]}{\text{sup}}\left\{\frac{1}{\varrho^{p+q}\Gamma \left(p+q\right)}\int_{1}^{t}e^{\left(\left(\varrho -1\right)/\varrho \right)\ln\left(t/u\right)}{\left(\text{ln}\frac{t}{u}\right)}^{p+q-1}\frac{\left|\xi \left(u,w_{1}\left(u\right)\right)-\xi \left(u,w_{2}\left(u\right)\right)\right|}{u}\text{d}u\right.\\ +\frac{\left|\lambda \right|}{\varrho^{p}\Gamma \left(p\right)}\int_{1}^{t}e^{\left(\left(\varrho -1\right)/\varrho \right)\ln\left(t/u\right)}{\left(\text{ln}\frac{t}{u}\right)}^{p-1}\frac{\left|w_{1}\left(u\right)-w_{2}\left(u\right)\right|}{u}\text{d}u\\ +\frac{e^{\left(\left(\varrho -1\right)/\varrho \right)\ln\;t}{\left|\ln\;t\right|}^{p+1}}{\varrho^{p}\left|\lambda -l-\varrho^{p}\Gamma \left(p+2\right)\right|}\left(\frac{\left|l-\lambda \right|}{\varrho^{q}\Gamma \left(p+q\right)}\int_{1}^{e}e^{\left(\left(\varrho -1\right)/\varrho \right)\ln\;u}{\left(1-\text{ln}u\right)}^{p+q-1}\frac{\left|\xi \left(u,w_{1}\left(u\right)\right)-\xi \left(u,w_{2}\left(u\right)\right)\right|}{u}\text{d}u\right.\\ +\frac{1}{\varrho^{q-p}\Gamma \left(q\right)}\int_{1}^{e}e^{\left(\left(\varrho -1\right)/\varrho \right)\ln\;u}{\left(1-\text{ln}u\right)}^{q-1}\frac{\left|\xi \left(u,w_{1}\left(u\right)\right)-\xi \left(u,w_{2}\left(u\right)\right)\right|}{u}\text{d}u\\ +\frac{\left|\lambda \right|\left|\lambda -l\right|}{\Gamma \left(p\right)}\int_{1}^{e}e^{\left(\left(\varrho -1\right)/\varrho \right)\ln\;u}{\left(1-\text{ln}u\right)}^{p-1}\frac{\left|w_{1}\left(u\right)-w_{2}\left(u\right)\right|}{u}\text{d}u\\ \left.+\frac{\left|l-\lambda +\varrho^{p}\Gamma \left(p+1\right)\right|}{\varrho^{\vartheta }\Gamma \left(p+1\right)\Gamma \left(\vartheta \right)}\int_{1}^{\eta }e^{\left(\left(\varrho -1\right)/\varrho \right)\left(\ln\;\eta -\ln\;u\right)}{\left(\ln\;\eta -\ln\;u\right)}^{\vartheta -1}\frac{\left|w_{1}\left(u\right)-w_{2}\left(u\right)\right|}{u}\text{d}u\right)\\ \left.+\frac{e^{\left(\left(\varrho -1\right)/\varrho \right)\ln\;t}{\left|\ln\;t\right|}^{p}}{\varrho^{p+\vartheta }\Gamma \left(p+1\right)\Gamma \left(\vartheta \right)}\int_{1}^{\eta }e^{\left(\left(\varrho -1\right)/\varrho \right)\left(\ln\;\eta -\ln\;u\right)}{\left(\ln\;\eta -\ln\;u\right)}^{\vartheta -1}\frac{\left|w_{1}\left(u\right)-w_{2}\left(u\right)\right|}{u}\text{d}u\right\}\\ \leq \left(\chi \left\|w_{1}-w_{2}\right\|\right)\underset{t\in \left[1,e\right]}{\sup}\left\{\frac{1}{\varrho^{p+q}\Gamma \left(p+q\right)}\int_{1}^{t}{\left(\ln\;t-\ln\;u\right)}^{p+q-1}\frac{\text{d}u}{u}\right.\\ +\frac{\left|l-\lambda \right|{\left|\ln\;t\right|}^{p+1}}{\varrho^{p+q}\left|\lambda -l-\varrho^{p}\Gamma \left(p+2\right)\right|\Gamma \left(p+1\right)}\int_{1}^{e}{\left(1-\ln\;u\right)}^{p+q-1}\frac{\text{d}u}{u}\\ \left.+\frac{{\left|\ln\;t\right|}^{p+1}}{\varrho^{q}\Gamma \left(q\right)\left|\lambda -l-\varrho^{p}\Gamma \left(p+2\right)\right|}\int_{1}^{e}{\left(1-\ln\;u\right)}^{q-1}\frac{\text{d}u}{u}\right\}\\ +\left\|w_{1}-w_{2}\right\|\underset{t\in \left[1,e\right]}{\sup}\left\{\frac{\left|\lambda \right|}{\varrho^{p}\Gamma \left(p\right)}\int_{1}^{t}{\left(\ln\;t-\ln\;u\right)}^{p-1}\frac{\text{d}u}{u}\right.\\ +\frac{\left|\lambda \right|\left|\lambda -l\right|{\left|\ln\;t\right|}^{p+1}}{\varrho^{p}\left|\lambda -l-\varrho^{p}\Gamma \left(p+2\right)\right|\Gamma \left(p\right)}\int_{1}^{e}{\left(1-\ln\;u\right)}^{p-1}\frac{\text{d}u}{u}\\ \frac{\left|l-\lambda +\varrho^{p}\Gamma \left(p+1\right)\right|{\left|\ln\;t\right|}^{p+1}}{\varrho^{\vartheta +p}\Gamma \left(p+1\right)\Gamma \left(\vartheta \right)\left|\lambda -l-\varrho^{p}\Gamma \left(p+2\right)\right|}\int_{1}^{\eta }{\left(\ln\;\eta -\ln\;u\right)}^{\vartheta -1}\frac{\text{d}u}{u}\\ \left.+\frac{{\left|\ln\;t\right|}^{p}}{\varrho^{p+\vartheta }\Gamma \left(p+1\right)\Gamma \left(\vartheta \right)}\int_{1}^{\eta }{\left(\ln\;\eta -\ln\;u\right)}^{\vartheta -1}\frac{\text{d}u}{u}\right\}\\ \leq \left(\chi \left\|w_{1}-w_{2}\right\|\right)\underset{t\in \left[1,e\right]}{\sup}\left\{\frac{1}{\varrho^{p+q}\Gamma \left(p+q+1\right)}+\frac{\left|l-\lambda \right|}{\varrho^{p+q}\left|\lambda -l-\varrho^{p}\Gamma \left(p+2\right)\right|\Gamma \left(p+q+1\right)}\right.\\ \left.+\frac{1}{\varrho^{q}\left|\lambda -l-\varrho^{p}\Gamma \left(p+2\right)\right|\Gamma \left(q+1\right)}\right\}\\ +\left\|w_{1}-w_{2}\right\|\left\{\frac{\left|\lambda \right|}{\varrho^{p}\Gamma \left(p+1\right)}+\frac{\left|\lambda \right|\left|\lambda -l\right|}{\varrho^{p}\left|\lambda -l-\varrho^{p}\Gamma \left(p+2\right)\right|\Gamma \left(p+1\right)}\right.\\ \left.\frac{\left|l-\lambda +\varrho^{p}\Gamma \left(p+1\right)\right|{\left(\ln\;\eta \right)}^{\vartheta }}{\varrho^{p+\vartheta }\Gamma \left(p+1\right)\Gamma \left(\vartheta +1\right)\left|\lambda -l-\varrho^{p}\Gamma \left(p+2\right)\right|}+\frac{{\left(\ln\;\eta \right)}^{\vartheta }}{\varrho^{p+\vartheta }\Gamma \left(p+1\right)\Gamma \left(\vartheta +1\right)}\right\}\\ \leq \left(\chi {ϒ}_{1}+{ϒ}_{2}\right)\left\|w_{1}-w_{2}\right\|, \end{array}$$

since *χ*ϒ_1_+ϒ_2_ < 1. Then, *𝔍* is a contractive operator. Hence, *𝔍* has a unique fixed point. As a result, there is only one solution for FLEs (15).

## 4. Stability Results

The HU stability of equation (15) is discussed in this section. The following is the definition of HU stability:

**Definition 8 .**

It is said that the integral equation (26) is HU stable. If ∃*ε*^*∗*^ ≥ 0 for some fixed constant *ζ*^*∗*^ > 0 satisfying the following:

(44) $$\begin{array}{c} |z\left(t\right)-\left(\frac{1}{\varrho^{p+q}\Gamma \left(p+q\right)}\int_{1}^{t}e^{\left(\left(\varrho -1\right)/\varrho \right)\left(\ln\;t-\ln\;u\right)}{\left(\ln\;t-\ln\;u\right)}^{p+q-1}\frac{\xi \left(u,z\left(u\right)\right)}{u}\text{d}u\right.\\ -\frac{\lambda }{\varrho^{p}\Gamma \left(p\right)}\int_{1}^{t}e^{\left(\left(\varrho -1\right)/\varrho \right)\left(\ln\;t-\ln\;u\right)}{\left(\ln\;t-\ln\;u\right)}^{p-1}\frac{z\left(u\right)}{u}\text{d}u\\ +\frac{e^{\left(\left(\varrho -1\right)/\varrho \right)\ln\;t}{\left(\ln\;t\right)}^{p+1}}{\varrho^{p}\left(\lambda -l-\varrho^{p}\Gamma \left(p+2\right)\right)}\left(\frac{l-\lambda }{\varrho^{q}\Gamma \left(p+q\right)}\int_{1}^{e}e^{\left(\left(\varrho -1\right)/\varrho \right)\ln\;u}{\left(1-\ln\;u\right)}^{p+q-1}\frac{\xi \left(u,z\left(u\right)\right)}{u}\text{d}u\right.\\ +\frac{1}{\varrho^{q-p}\Gamma \left(q\right)}\int_{1}^{e}e^{\left(\left(\varrho -1\right)/\varrho \right)\ln\;u}{\left(1-\ln\;u\right)}^{q-1}\frac{\xi \left(u,z\left(u\right)\right)}{u}\text{d}u-\frac{\lambda \left(\lambda -l\right)}{\Gamma \left(p\right)}\int_{1}^{e}e^{\left(\left(\varrho -1\right)/\varrho \right)\ln\;u}{\left(1-\ln\;u\right)}^{p-1}\frac{z\left(u\right)}{u}\text{d}u\\ +\frac{l-\lambda +\varrho^{p}\Gamma \left(p+1\right)}{\varrho^{\vartheta }\Gamma \left(p+1\right)\Gamma \left(\vartheta \right)}\int_{1}^{\eta }e^{\left(\left(\varrho -1\right)/\varrho \right)\left(\ln\;\eta -\ln\;u\right)}{\left(\ln\;\eta -\ln\;u\right)}^{\vartheta -1}\frac{z\left(u\right)}{u}\text{d}u\\ \left.+\frac{e^{\left(\left(\varrho -1\right)/\varrho \right)\ln\;t}{\left(\ln\;t\right)}^{p}}{\varrho^{p+\vartheta }\Gamma \left(p+1\right)\Gamma \left(\vartheta \right)}\int_{1}^{\eta }e^{\left(\left(\varrho -1\right)/\varrho \right)\left(\ln\;\eta -\ln\;u\right)}{\left(\ln\;\eta -\ln\;u\right)}^{\vartheta -1}\frac{z\left(u\right)}{u}\text{d}u\right)|\leq \zeta^{*}, \end{array}$$

then there exists a continuous function *z*_1_(*t*) which satisfies

(45) $$\begin{array}{c} z_{1}\left(t\right)=\frac{1}{\varrho^{p+q}\Gamma \left(p+q\right)}\int_{1}^{t}e^{\left(\left(\varrho -1\right)/\varrho \right)\left(\ln\;t-\ln\;u\right)}{\left(\ln\;t-\ln\;u\right)}^{p+q-1}\frac{\xi \left(u,z_{1}\left(u\right)\right)}{u}\text{d}u\\ -\frac{\lambda }{\varrho^{p}\Gamma \left(p\right)}\int_{1}^{t}e^{\left(\left(\varrho -1\right)/\varrho \right)\left(\ln\;t-\ln\;u\right)}{\left(\ln\;t-\ln\;u\right)}^{p-1}\frac{z_{1}\left(u\right)}{u}\text{d}u\\ +\frac{e^{\left(\left(\varrho -1\right)/\varrho \right)\ln\;t}{\left(\ln\;t\right)}^{p+1}}{\varrho^{p}\left(\lambda -l-\varrho^{p}\Gamma \left(p+2\right)\right)}\left(\frac{l-\lambda }{\varrho^{q}\Gamma \left(p+q\right)}\int_{1}^{e}e^{\left(\left(\varrho -1\right)/\varrho \right)\ln\;u}{\left(1-\ln\;u\right)}^{p+q-1}\frac{\xi \left(u,z_{1}\left(u\right)\right)}{u}\text{d}u\right.\\ +\frac{1}{\varrho^{q-p}\Gamma \left(q\right)}\int_{1}^{e}e^{\left(\left(\varrho -1\right)/\varrho \right)\ln\;u}{\left(1-\ln\;u\right)}^{q-1}\frac{\xi \left(u,z_{1}\left(u\right)\right)}{u}\text{d}u-\frac{\lambda \left(\lambda -l\right)}{\Gamma \left(p\right)}\int_{1}^{e}e^{\left(\left(\varrho -1\right)/\varrho \right)\ln\;u}{\left(1-\ln\;u\right)}^{p-1}\frac{z_{1}\left(u\right)}{u}\text{d}u\\ +\frac{l-\lambda +\varrho^{p}\Gamma \left(p+1\right)}{\varrho^{\vartheta }\Gamma \left(p+1\right)\Gamma \left(\vartheta \right)}\int_{1}^{\eta }e^{\left(\left(\varrho -1\right)/\varrho \right)\left(\ln\;\eta -\ln\;u\right)}{\left(\ln\;\eta -\ln\;u\right)}^{\vartheta -1}\frac{z_{1}\left(u\right)}{u}\text{d}u\\ +\frac{e^{\left(\left(\varrho -1\right)/\varrho \right)\ln\;t}{\left(\ln\;t\right)}^{p}}{\varrho^{p+\vartheta }\Gamma \left(p+1\right)\Gamma \left(\vartheta \right)}\int_{1}^{\eta }e^{\left(\left(\varrho -1\right)/\varrho \right)\left(\ln\;\eta -\ln\;u\right)}{\left(\ln\;\eta -\ln\;u\right)}^{\vartheta -1}\frac{z_{1}\left(u\right)}{u}\text{d}u, \end{array}$$

such that

(46) $$\begin{array}{c} \left|z\left(t\right)-z_{1}\left(t\right)\right|\leq \varepsilon^{*}\zeta^{*}. \end{array}$$

**Theorem 4 .**

Let us imagine that the continuous function *ξ* : [1, *e*] × *ℝ*⟶*ℝ* satisfies inferences (I) and (II), then equation (15) is HU stable.

**Proof**

(47) $$\begin{array}{c} \left|z\left(t\right)-z_{1}\left(t\right)\right|\leq \text{sup}\left\{\frac{1}{\varrho^{p+q}\Gamma \left(p+q\right)}\int_{1}^{t}e^{\left(\left(\varrho -1\right)/\varrho \right)\left(\ln\;t-\ln\;u\right)}{\left(\ln\;t-\ln\;u\right)}^{p+q-1}\frac{\left|\xi \left(u,z\left(u\right)\right)-\xi \left(u,z_{1}\left(u\right)\right)\right|}{u}\text{d}u\right.\\ -\frac{\left|\lambda \right|}{\varrho^{p}\Gamma \left(p\right)}\int_{1}^{t}e^{\left(\left(\varrho -1\right)/\varrho \right)\left(\ln\;t-\ln\;u\right)}{\left(\ln\;t-\ln\;u\right)}^{p-1}\frac{\left|z\left(u\right)-z_{1}\left(u\right)\right|}{u}\text{d}u\\ +\frac{e^{\left(\left(\varrho -1\right)/\varrho \right)\ln\;t}{\left(\ln\;t\right)}^{p+1}}{\varrho^{p}\left(\lambda -l-\varrho^{p}\Gamma \left(p+2\right)\right)}\left(\frac{l-\lambda }{\varrho^{q}\Gamma \left(p+q\right)}\int_{1}^{e}e^{\left(\left(\varrho -1\right)/\varrho \right)\ln\;u}{\left(1-\ln\;u\right)}^{p+q-1}\frac{\left|\xi \left(u,z\left(u\right)\right)-\xi \left(u,z_{1}\left(u\right)\right)\right|}{u}\text{d}u\right.\\ +\frac{1}{\varrho^{q-p}\Gamma \left(q\right)}\int_{1}^{e}e^{\left(\left(\varrho -1\right)/\varrho \right)\ln\;u}{\left(1-\ln\;u\right)}^{q-1}\frac{\left|\xi \left(u,z\left(u\right)\right)-\xi \left(u,z_{1}\left(u\right)\right)\right|}{u}\text{d}u\\ -\frac{\lambda \left(\lambda -l\right)}{\Gamma \left(p\right)}\int_{1}^{e}e^{\left(\left(\varrho -1\right)/\varrho \right)\ln\;u}{\left(1-\ln\;u\right)}^{p-1}\frac{\left|z\left(u\right)-z_{1}\left(u\right)\right|}{u}\text{d}u\\ +\left.\frac{l-\lambda +\varrho^{p}\Gamma \left(p+1\right)}{\varrho^{\vartheta }\Gamma \left(p+1\right)\Gamma \left(\vartheta \right)}\int_{1}^{\eta }e^{\left(\left(\varrho -1\right)/\varrho \right)\left(\ln\;\eta -\ln\;u\right)}{\left(\ln\;\eta -\ln\;u\right)}^{\vartheta -1}\frac{\left|z\left(u\right)-z_{1}\left(u\right)\right|}{u}\text{d}u\right)\\ \left.+\frac{e^{\left(\left(\varrho -1\right)/\varrho \right)\ln\;t}{\left(\ln\;t\right)}^{p}}{\varrho^{p+\vartheta }\Gamma \left(p+1\right)\Gamma \left(\vartheta \right)}\int_{1}^{\eta }e^{\left(\left(\varrho -1\right)/\varrho \right)\left(\ln\;\eta -\ln\;u\right)}{\left(\ln\;\eta -\ln\;u\right)}^{\vartheta -1}\frac{\left|z\left(u\right)-z_{1}\left(u\right)\right|}{u}\text{d}u\right\}\\ \leq \left(\chi \left\|z-z_{1}\right\|\right)\underset{t\in \left[1,e\right]}{\text{sup}}\left\{\frac{1}{\varrho^{p+q}\Gamma \left(p+q\right)}\int_{1}^{t}{\left(\ln\;t-\ln\;u\right)}^{p+q-1}\frac{\text{d}u}{u}\right.\\ +\frac{\left|l-\lambda \right|{\left|\text{ln}t\right|}^{p+1}}{\varrho^{p+q}\left|\lambda -l-\varrho^{p}\Gamma \left(p+2\right)\right|\Gamma \left(p+1\right)}\int_{1}^{e}{\left(1-\ln\;u\right)}^{p+q-1}\frac{\text{d}u}{u}\\ \left.+\frac{{\left|\ln\;t\right|}^{p+1}}{\varrho^{q}\Gamma \left(q\right)\left|\lambda -l-\varrho^{p}\Gamma \left(p+2\right)\right|}\int_{1}^{e}{\left(1-\ln\;u\right)}^{q-1}\frac{\text{d}u}{u}\right\}\\ +\left\|z-z_{1}\right\|\underset{t\in \left[1,e\right]}{\text{sup}}\left\{\frac{\left|\lambda \right|}{\varrho^{p}\Gamma \left(p\right)}\int_{1}^{t}{\left(\ln\;t-\ln\;u\right)}^{p-1}\frac{\text{d}u}{u}\right.+\frac{\left|\lambda \right|\left|\lambda -l\right|{\left|\ln\;t\right|}^{p+1}}{\varrho^{p}\left|\lambda -l-\varrho^{p}\Gamma \left(p+2\right)\right|\Gamma \left(p\right)}\int_{1}^{e}{\left(1-\ln\;u\right)}^{p-1}\frac{\text{d}u}{u}\\ +\frac{\left|l-\lambda +\varrho^{p}\Gamma \left(p+1\right)\right|{\left|\ln\;t\right|}^{p+1}}{\varrho^{\vartheta +p}\Gamma \left(p+1\right)\Gamma \left(\vartheta \right)\left|\lambda -l-\varrho^{p}\Gamma \left(p+2\right)\right|}\int_{1}^{\eta }{\left(\ln\;\eta -\ln\;u\right)}^{\vartheta -1}\frac{\text{d}u}{u}\\ \left.+\frac{{\left|\ln\;t\right|}^{p}}{\varrho^{p+\vartheta }\Gamma \left(p+1\right)\Gamma \left(\vartheta \right)}\int_{1}^{\eta }{\left(\ln\;\eta -\ln\;u\right)}^{\vartheta -1}\frac{\text{d}u}{u}\right\}\\ \leq \left(\chi \left\|z-z_{1}\right\|\right)\underset{t\in \left[1,e\right]}{\text{sup}}\left\{\frac{1}{\varrho^{p+q}\Gamma \left(p+q+1\right)}+\frac{\left|l-\lambda \right|}{\varrho^{p+q}\left|\lambda -l-\varrho^{p}\Gamma \left(p+2\right)\right|\Gamma \left(p+q+1\right)}\right.\\ \left.+\frac{1}{\varrho^{q}\left|\lambda -l-\varrho^{p}\Gamma \left(p+2\right)\right|\Gamma \left(q+1\right)}\right\}+\left\|z-z_{1}\right\|\left\{\frac{\lambda }{\varrho^{p}\Gamma \left(p+1\right)}+\frac{\left|\lambda \right|\left|\lambda -l\right|}{\varrho^{p}\left|\lambda -l-\varrho^{p}\Gamma \left(p+2\right)\right|\Gamma \left(p+1\right)}\right.\\ \left.+\frac{\left|l-\lambda +\varrho^{p}\Gamma \left(p+1\right)\right|{\left(\text{ln}\eta \right)}^{\vartheta }}{\varrho^{p+\vartheta }\Gamma \left(p+1\right)\Gamma \left(\vartheta +1\right)\left|\lambda -l-\varrho^{p}\Gamma \left(p+2\right)\right|}+\frac{{\left(\ln\;\eta \right)}^{\vartheta }}{\varrho^{p+\vartheta }\Gamma \left(p+1\right)\Gamma \left(\vartheta +1\right)}\right\}\\ \leq \left(\chi {ϒ}_{1}+{ϒ}_{2}\right)\left\|z-z_{1}\right\|. \end{array}$$

Hence, by using equation (47), equation (26) is HU stable. Therefore, the FLEs (15) are HU stable.

## 5. An Example

Let us take a look at the following FLEs:

(48) $$\begin{array}{c} \left\{\begin{array}{c} {}^{\mathrm{CHP}}D^{2.5,0.5}\left({}^{\mathrm{CHP}}D^{1.5,0.5}+\frac{1}{50}\right)z\left(t\right)=\xi \left(t,z\left(t\right)\right),\;t\in \left[1,e\right],\\ z\left(1\right)=0,\\ z^{\prime }\left(1\right)=0,\\ z^{\prime \prime }\left(1\right)=0,\\ {}^{\mathrm{CHP}}D^{1.5,0.5}z\left(1\right)={ℷ}^{4,0.5}z\left(\frac{e}{2}\right),\\ {}^{\mathrm{CHP}}D^{1.5,0.5}z\left(e\right)+\frac{1}{4}z\left(e\right)=0. \end{array}\right. \end{array}$$

Choose

(49) $$\begin{array}{c} \left|\xi \left(t,z\left(t\right)\right)\right|=\frac{1}{t+25}\left(\frac{\left|z\right|}{\left|z\right|+10}\right). \end{array}$$

It is obvious that the prerequisites (I1), (I2), and (I3) are fulfilled.

By putting *χ*=(1/4), we get ϒ_3_=0.372486 < 1, ϒ_2_=0.0565441, and ϒ_1_=1.98731. Hence, all assumptions of Theorem 2 are met. As a result of Theorem 2, there must be at least one solution to equation (15).

On the other hand, *χ*ϒ_1_+ϒ_2_=0.553373 < 1, this implies that Theorem 3 demonstrates that equation (15) has only one solution. The requirements for HU stability can also be found in a simple manner. Equation (15) is HU stable, so we conclude.

## 6. Conclusion

Fractional Langevin equations have a considerable role in modeling varied physical phenomena. For instance, they have been employed for describing single-file prevalence [19] and the conduct of unshackled particles driven by internal noises [16]. The present paper examined the fractional Langevin equations with generalized proportional Hadamard–Caputo derivative of different orders. Nonlocal integrals and nonperiodic boundary conditions have been deemed as well. With the assistance of Krasnoselskii and Banach fixed point theorems, existence, uniqueness, and Hyres–Ulam stability for the solution have been amply investigated. Moreover, an application example has been offered to reinforce the accuracy of the gained outcomes.

## Data Availability

The data that support the findings of this study are available from the corresponding author upon reasonable request.
